# High-Internal-Phase Pickering Emulsions Stabilized by β‑Lactoglobulin Nanoparticles: Effects of Rheology, Stability, and Aging

**DOI:** 10.1021/acs.langmuir.6c04106

**Published:** 2026-10-22

**Authors:** Rini P. Ravindranathan, Geir Helgesen, Matti Knaapila, Ann Terry, Jon Otto Fossum, Kenneth Dahl Knudsen

**Affiliations:** † Institute for Energy Technology, Instituttveien 18, 2007 Kjeller, Norway; ‡ Norwegian University of Science and Technology, Høgskoleringen 1, 7034 Trondheim, Norway; § MAX IV Laboratory, Fotongatan 2, 224 84 Lund, Sweden

## Abstract

High-internal-phase Pickering emulsions have found a greater potential in food industry. β-Lactoglobulin (BLG) is a major component of whey protein and is of interest due to its easy availability, nutritional benefits, and potentially stabilizing properties. Here, we present our work on Pickering high-internal-phase emulsions (HIPEs) with a dispersed-phase oil fraction of 75 wt %, stabilized using BLG nanoparticles. These nanoparticles were prepared using an antisolvent precipitation technique, eliminating the need for any external cross-linking agent. The present work focuses on stability of Pickering HIPEs, specifically on the effects of particle and salt concentration, by employing techniques like small-angle X-ray scattering along with scanning electron microscopy, optical microscopy, light scattering, and rheology. Given that Pickering HIPEs exhibit marked viscoelastic properties, we also employ RheoSAXS to elucidate structural changes of the emulsion under shear. We show that higher particle concentrations (3–5 wt %) can enhance the stability of the emulsions. Furthermore, by measuring the emulsions under different physical conditions (fresh, aged, and aged at elevated temperatures), we show that aging (shelf storage) over a period of 90 days can induce structural changes in the system. Here, we employ combinations of scattering models to understand the structural properties of the emulsions. Our studies show that SAXS, along with rheology, is an effective tool for studying these emulsions with high oil concentrations, providing insight into the structure and behavior of food-grade systems.

## Graphical abstract

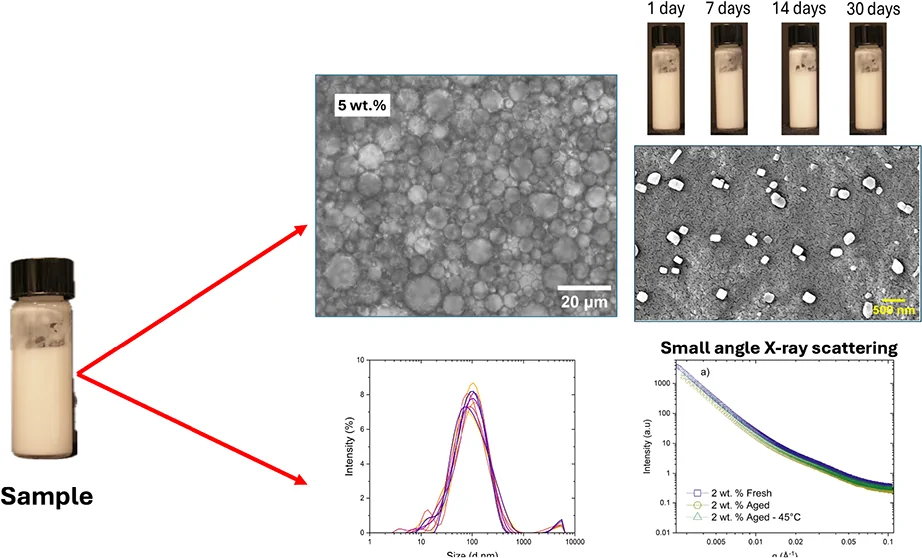

## 1 Introduction

A wide range of food products come under the category of emulsions, which are a mixture of two immiscible liquids. Pickering emulsions are a class of emulsions where droplets are stabilized by solid particles attached irreversibly to the interface. Pickering emulsions typically show improved stability, and their biocompatibility and potential health benefits have led to increased interest during recent years in food-grade materials prepared with Pickering particles.^1,2^ High-internal-phase emulsions are a particular class of emulsions defined by the volume fraction of the dispersed phase (>74%). The high concentration of the internal phase leads to a crowded packing of the droplets, which strongly affects the rheological and structural properties of the emulsions, e.g., a high viscosity leading to a solid-like behavior and high yield stress.^3^ This high concentration sometimes leads to nonspherical droplets due to the packing constraints.^4,5^

Generally, high-internal-phase emulsions have been prepared using conventional surfactants. However, a variant of this, HIPEs, have gained attention recently due to the advantages of being eco-friendly, having high stability, and promising applications in food science,^6^ pharmaceutics, and cosmetics. These emulsions typically consist of very-high-volume fractions, above 74% since the polydispersity in droplet size allows for higher concentrations than in the theoretical situation with a monodisperse system, together with the fact that the droplets can deform to some extent.^7^ Here, Pickering particles adsorbed to the droplet surface prevent coalescence, and the high-volume fractions result in tightly packed or jammed droplets, which contribute to a solid-like behavior.

As examples of such systems, Pickering HIPEs have been made using, e.g., whey-protein-based nanoparticles combined with curcumin and different amounts of proanthocyanins.^8,9^ Pickering HIPEs prepared with flaxseed particles have demonstrated improved emulsion stability, and it was additionally found that adding these Pickering emulsions into beef as a substitute for animal fat improved the quality of the meat significantly without destroying aroma or color.^10,8^ Factors such as particle size, wettability, and surface charge are relevant for achieving a sufficiently stable emulsion.^11–14^ It is estimated that 40% of the cost of developing a new food emulsion is incurred in shelf life testing, where the major criterion is the onset of the visual nonuniformity.^15^ Thus, emulsions and suspensions are susceptible to instabilities that affect the shelf life of the material. Besides, studies have shown that adding salt or surfactant to the Pickering emulsions can alter the structure and stability of the systems.^16^ Dai et al. studied the freeze–thaw stability and heating stability of BLGNP-stabilized high-internal-phase Pickering emulsions.^17–19^

Pickering particles can be produced from proteins by different methods like antisolvent precipitation^20,21^ solvent evaporation^22^ and heating, or solvent-induced aggregates or microgels.^23^ Antisolvent precipitation involves mixing a protein dispersion with an organic solvent at a pH far from the isoelectric point and then using vortex mixing or ultrasonication. For hydrophilic proteins, an organic solvent like ethanol or acetone is used as the antisolvent.^17,24,25^ For proteins that have less water solubility, the antisolvent will normally be water.^26^ β-Lactoglobulin (BLG) is found in the milk of mammals and is a major component of whey protein as well.^27^ Various studies have been conducted where BLG proteins with or without modification are used as stabilizers.^28^ BLG proteins can be made into BLG nanoparticles (BLGNP) using chemical cross-linking agents like glutaraldehyde and formaldehyde, but their application in food systems is limited due to its toxicity. Ko et al. prepared sub-100 nm BLGNPs using the desolvation method where these particles were cross-linked using glutaraldehyde.^24,25^ Dai et al. prepared and investigated Pickering HIPEs stabilized by BLGNP.^17^ Here Genipin was used as a cross-linking agent instead of glutaraldehyde due to the severe toxicity of the latter. Even though Genipin is a natural cross-linking agent, the safety data sheet according to Regulation (EC) No. 1907/2006 classifies it as a category-3 toxic substance (toxic if swallowed). But it has been shown that it is possible to prepare BLGNPs using heat denaturation without a cross-linker.^29^ In our previous work, we discussed Pickering emulsions stabilized with BLGNPs at different oil concentrations (40–70 wt %).^30^ In the present work, we enter the high range and prepare Pickering HIPEs at 75 wt % oil, with sunflower oil as the dispersed phase. The BLGNPs used for stabilization were prepared by applying the antisolvent precipitation technique without adding any external cross-linking agent, like in our previous study.

Most of these studies have been conducted using commonly available techniques such as microscopy (optical, scanning electron microscopy), stability testing, light scattering, and rheology. Small-angle scattering, especially small-angle X-ray scattering, has gained its popularity because of its ability to provide information on shape, size, structure, and particle interactions from nanoscale to near-microscale within a very short interval of time.^31,32^ Using a high-flux synchrotron source, SAXS is a very powerful tool to analyze the emulsions in situ without destruction of the samples (by using a short exposure time). This technique also has the potential to effectively study the structural evolution of emulsions over time when exposed to extended shelf storage or high temperatures. Only a few systematic studies have been conducted on emulsions, especially high-internal-phase emulsions, using SAXS.

Larson-Smith et al. used small-angle scattering (X-rays and neutrons) to study Pickering emulsions stabilized with silica particles.^33^ Here, the self-correlation of the spherical core of oil droplets and adsorbed particles at the interface, along with the particle–particle and core–particle correlation terms, was considered using the raspberry model. This idealized model, however, is not fully adequate to describe the behavior of food-grade Pickering particles, such as BLGNP, which are made by desolvation and precipitation. Reynolds et al. conducted a series of small-angle scattering experiments on high-internal-phase water-in-oil emulsions stabilized by surfactants, using neutrons (SANS) as the probe.^32,34–39^ Heiden-Hecht et al. studied BLG protein-stabilized emulsions with low protein concentrations using small-angle scattering techniques, describing the protein structure at the interface.^28^ In this case, interparticle interactions (structure factor) were not necessary to take into consideration because of the very low protein concentrations (<0.75%). Further, Olsmats et al. used SAXS and USAXS techniques to study the structure of emulsions stabilized with pea protein at higher concentrations and up to about 50% oil.^40^

The goal of the present study is to elucidate the internal structure and stability properties of high-internal-phase Pickering emulsions stabilized using *non-cross-linked* BLGNPs by using small-angle X-ray scattering as the main technique. We investigate the effect of particle and salt concentrations on these emulsions as well as how they undergo changes over a longer period of time and at a high temperature. Additionally, we employ visual observation, light scattering, and scanning electron microscopy (SEM) to examine the Pickering particles and perform rheology, viscosity, and elastic modulus measurements on the emulsions. Our results show that BLG nanoparticles can be prepared without incorporating any toxic cross-linking agents and that these particles can be used for preparing Pickering HIPEs with varying particle and salt concentrations. Next, we study the structural evolution of these emulsions with aging and high temperature. Finally, the internal structure of the materials under deformations such as shear and strain was investigated by using rheoSAXS (rheology-coupled small-angle X-ray scattering) measurements.

## 2 Experimental Methods

### 2.1 Materials

β-lactoglobulin from bovine milk (BLG) was obtained from Sigma-Aldrich and used without further purification. The sunflower oil used was purchased from a local supermarket and used as is instantly after opening. Absolute ethanol (≥99.7%) was obtained from VWR chemicals. All the chemical solvents used in the study were of analytical grade and supplied by Sigma-Aldrich.

### 2.2 Preparation of BLGNPs

BLG nanoparticles were prepared by a modified desolvation technique,^17,24^ as described in ref 30. Initially, 3 wt % BLG proteins were dissolved in 10 mM NaCl solution. The dispersion was stored overnight in a refrigerator at 4 °C to allow complete hydration of the proteins. Prior to desolvation, the dispersion was passed through a 0.22 μm filter, which removes any undissolved particulates or bigger aggregates. The filtered dispersion was then subjected to heat treatment in a preheated water bath maintained at 60 °C for 30 min. The pH of the solution was gradually adjusted to 9 using an NaOH solution. Absolute ethanol was added dropwise as a desolvation agent, at a rate of 1 mL/min while continuously stirring, until the solution became visibly turbid. Approximately 22 mL of absolute ethanol was introduced into the solution. After adding ethanol, the final pH at the precipitation stage was approximately 9.4–9.5. The rate of ethanol addition and pH strongly affects the size of the particles. After pH adjustment and desolvation, the resultant precipitate was centrifuged at 10,000 rpm for 30 min. The precipitate was then used to prepare Pickering emulsions.

### 2.3 Scanning Electron Microscopy

SEM images of BLGNPs were taken with a field-emission scanning electron microscope (FEI Apreo) using an operating voltage of 4 kV. The BLGNP dispersions, diluted with distilled water, were deposited onto a silicon wafer and allowed to dry at room temperature, followed by gold coating via electrospraying for SEM imaging.

### 2.4 Dynamic Light Scattering

The particle size and zeta potential of BLGNP dispersions were studied using a DLS particle size analyzer (Nano ZS, Malvern Instruments Ltd., UK) at 25 °C, employing a He–Ne laser with a 173° scattering angle. Before the measurements, BLGNPs were dispersed in 10 mL of distilled water adjusted to pH 9.0 with NaOH. These suspensions were stirred continuously at room temperature for 12 h. The zeta potential was determined using DTS1070 cells. Each sample underwent at least 10 repetitions and were checked for reproducibility.

### 2.5 Emulsion Preparation

BLGNP solutions with various w/v concentrations were stirred at room temperature in solutions with different molar concentrations overnight. Then, different volumes of sunflower oil were added to the suspension, and they were immediately homogenized at a speed of 17,000 rpm for 3 min (Benchmark model D100 hand-held homogenizer). Consequently, the emulsions were stored in an airtight glass vial at room temperature for a longer period of time.

### 2.6 Optical Microscopy

The HIPEs were examined using a Leica DMB upright microscope at a magnification of 100×. To prepare the sample, a small amount of emulsion (0.1 mL) was placed on a glass microscope slide and gently covered with a coverslip to form a thin layer. Images were captured using a digital camera mounted on a microscope with software installed on the connected computer (uEye Cockpit).

### 2.7 Rheometry

Viscosity and amplitude sweep studies were performed on the emulsions using a modular compact rheometer (Anton Paar MCR 300) with 20 mm diameter parallel plates at 25 °C. After 180 s of waiting time to ensure a steady state, the apparent viscosity of the emulsions was recorded as a function of shear rate (0.01–1000 s^–1^). The dynamic viscoelastic properties of the emulsions were characterized using amplitude sweep mode. Frequency sweep measurements were conducted at a constant frequency of 0.1 Hz, with the strain increasing gradually from 0.1 to 100.

### 2.8 SAXS

SAXS measurements were conducted on emulsions and dispersions under varying particles and salt concentrations. Emulsions were also measured under three different conditions: Fresh, Aged, and Aged-HT. Fresh HI-BLGNPem refers to emulsions examined immediately following homogenization. Aged HI-BLGNPem represent samples measured two months after storage at room temperature, during which phase separation has occurred. Additionally, aged emulsions subjected to high temperatures (Aged HI-BLGNPem HT) were measured at 45 °C. All samples were initially prepared in glass vials and were transferred and measured in a glass capillary (1.5 mm inner diameter) and were measured at both room temperature and 45 °C.

Measurements on freshly prepared emulsions were made at the beamline BM26 (DUBBLE) at the European Synchrotron Radiation Facility (ESRF). The X-ray used was of 1.03 Å wavelength, with a sample-to-detector distance of 9.342 m, giving a usable *q* range from 0.002 Å^–1^ to 0.14 Å^–1^. To ensure consistent initial conditions, the emulsions were homogenized just before the measurements. The data were collected with a 2D Pilatus 1 M detector, and analyses were performed using the software installed at the beamline. Aged emulsions were measured at the Co-SAXS beamline at the MAX IV synchrotron facility, using a 1 Å wavelength beam, with a *q* range of 0.0025–0.2 Å^–1^. The measurements were taken two months after the sample preparation, where samples with less stability had undergone phase separation. The measurements were focused on the phase-separated emulsion-rich layer (see Figure 4). The samples were measured at 25 and 45 °C. Data analysis and reduction were performed in Jupyter Notebook on a local computer at MAX IV, with model fitting carried out using SasView software (SasView 5.0.6, 2024). A core–shell sphere model was used to describe the scattering from the oil droplet plus interface region and for the BLG particles, an elliptical model was used (to allow for deviations from an average spherical shape). The latter included a structure factor (Hayter–Penfold MSA) to account for interparticle interactions due to the surface charge. Initially, measurements on BLG particle dispersions (no oil) were performed, and in this case, the elliptical particle model was used in combination with a c model to describe the scattering at low values of the scattering vector *q*.

RheoSAXS measurements were performed at the Co-SAXS beamline at the MAX IV laboratory. Viscosity measurements were carried out on the emulsions at the beamline using a rheometer (Anton Paar MCR702) equipped with plate–plate geometry integrated into the beamline setup. The X-ray beam was oriented perpendicular to the shear plane. SAXS measurements were obtained simultaneously during the application of the shear. The scattering profiles were obtained for the emulsions applied before shear and during the maximum shear (1000 s^–1^) to probe structural changes during the flow.

For data analysis, the Guinier–Porod model combined with ellipsoid and Hayter–Penfold MSA as structure factor is used to describe scattering from Pickering particles as point-like charges. For HIPEs, core–shell sphere model is combined with ellipsoid and Hayter–Penfold MSA is used. Core–shell sphere model represents the scattering from average oil droplet with an interface layer on top and ellipsoid model takes scattering from protein particles dispersed in the aqueous phase. The Hayter–Penfold MSA takes interparticle interactions into account. The scattering length density (SLD) was set to 11.6 × 10^–6^ Å^–2^ for the protein particles and to 9.41 × 10^–6^ Å^–2^ for the water solvent.

We refer to our previous work^30^ concerning the general form of the scattering intensity.^42–44^ Briefly, for a core–shell sphere model, the 1D scattering intensity can be presented as a function of the scattering vector *q* ^41,42^

1 $$I\left(q\right)=\frac{scale}{V}F^{2}\left(q\right)+b$$

where

2 $$F\left(q\right)=3V_{c}\left(\rho_{c}-\rho_{s}\right)\frac{sin\left(qr_{c}\right)-qr_{c}cos\left(qr_{c}\right)}{{\left(qr_{c}\right)}^{3}}+3V\left(\rho_{s}-\rho_{solv}\right)\frac{\sin\left(qr_{s}\right)-qr_{s}\cos\left(qr_{s}\right)}{{\left(qr_{s}\right)}^{3}}$$

Here, *V* and *V*_*c*_ are the volumes of the whole particle and volume of the core, respectively, *r*_*c*_ and *r*_*s*_ are the radii of the core and shell, respectively, *r*_*s*_ is the sum of the core radius and the layer thickness, i.e., the overall radius of the particle, and ρ_*c*_, ρ_*s*_, and ρ_solv_ are the scattering length densities (SLD) of the core, shell, and solvent. The parameter *b* is an incoherent flat background.^30^

For oblate ellipsoids, the scattering intensity function for oriented ellipsoids is given by^43,44^

3 $$I\left(q\right)=\frac{scale}{V}F^{2}\left(q,\alpha \right)+b$$

where

4 $$F\left(q,\alpha \right)=\Delta \rho V\frac{3\sin\mathrm{qr}-\mathrm{qr}\cos\mathrm{qr}}{{\left(\mathrm{qr}\right)}^{3}}$$

with

5 $$r={\left[R_{eq}^{2}sin^{2}\alpha +R_{p}^{2}cos^{2}\alpha \right]}^{1/2}$$

where α is the angle between the axis of the ellipsoid and *q*. *V* = (4/3)­π*R*_*p*_ *R*_eq_^2^ is the volume of the ellipsoid, *R*_*p*_ and *R*_eq_ are the polar and equatorial radii perpendicular to the rotational axis of the ellipsoid, and Δρ is the difference between the scattering length density of the particle and the solvent.^30^

Finally, the scattering intensity from a system described by Guinier–Porod model can be written as^45–47^

6 $$\begin{array}{l} I\left(q\right)=\frac{G}{q^{s}}\exp\left(\frac{-q^{2}R_{g}^{2}}{3-s}\right),q\leq q_{1}\\ \frac{D}{q^{m}},q\geq q_{1} \end{array}$$

Here, *R*_*g*_ is the radius of gyration and *G* and *D* are scaling constants. The parameter 3-*s* represent dimensionality. For 3D globular objects, *s* = 0. Here, *m* is the experimentally observed exponent and *q*_1_ is the value of *q* where the Guinier and Porod regions overlap.

## 3 Results and Discussion

### 3.1 Particle Morphology

The morphology and surface characteristics of BLGNPs were investigated by using SEM. As shown in Figure 1, BLGNPs exhibit a heterogeneous, yet tendency toward an asymmetric shape with occasionally irregular structures. The BLGNPs were prepared via an antisolvent precipitation technique as described in Section 2. The particle sizes were observed to be within the 50–300 nm range. However, these values should be regarded as qualitative estimates rather than statistically derived particle size distributions. The observed variation may be associated with the antisolvent precipitation process and possible aggregation during sample preparation for SEM. This size distribution is favorable for Pickering emulsion stabilization, where particles adsorb to the oil–water interface.^17,48,49^ The irregular morphologies and variability in the particle size observed in some particles could be attributed to the solvent precipitation technique. The surfaces of the particles appear to be relatively smooth without significant features or porosity. While large areas of the SEM image show well-dispersed particles, some regions exhibit clustering and partial aggregation. The particle aggregation could arise as a result of either desolvation or drying during sample preparation for SEM. Overall, the observed size distribution, shape, and dispersions should be favorable for Pickering emulsion stabilization. The particle shape can play an important role in stabilization since higher anisotropy can increase the desorption energy.^50^ The nanoscale size, along with a mild particle aggregation, helps in forming a densely packed interfacial layer suitable for stabilization.

**Figure 1:**
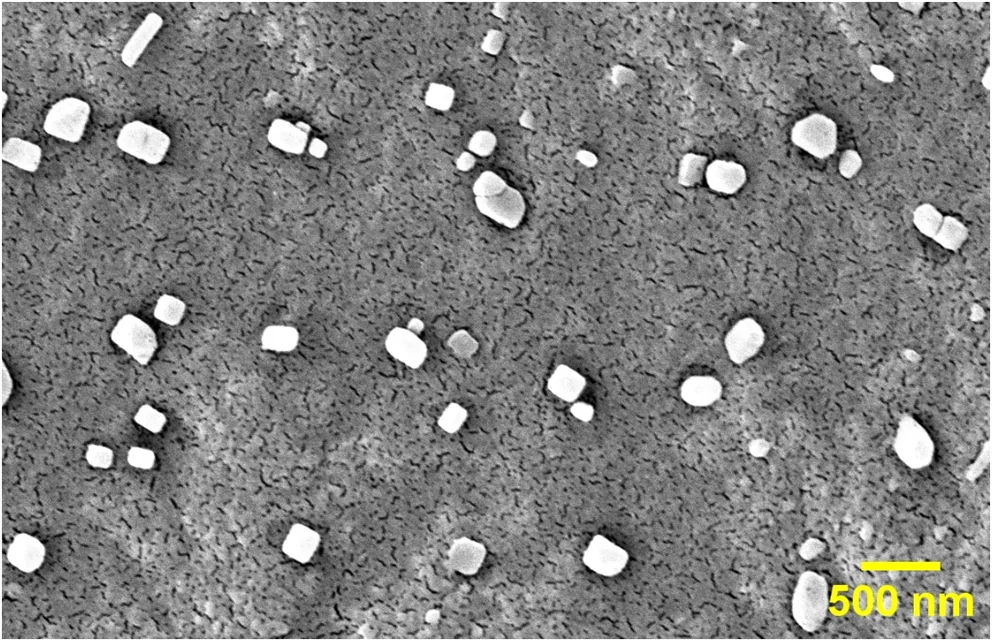
SEM image of BLGNP prepared via antisolvent precipitation. The nanoparticles appear with irregular morphologies and with a size range of 50–300 nm.

### 3.2 Particle Size and Zeta Potential

DLS measurements were primarily conducted to determine the hydrodynamic size (diameter) and polydispersity of 1 wt % BLGNPs dispersed in distilled water at pH 9 prior to emulsion measurements. The samples were measured at room temperature with the viscosity of water taken as 0.89 cP and its refractive index as 1.33.^51^ The polydispersity index was approximately 0.4, indicating a moderate degree of particle heterogeneity, likely due to the aggregation of the particles in the solution. Figure 2 shows the intensity-weighted and volume-weighted size distribution of BLGNPs. The intensity-weighed distribution (Figure 2a) indicates a peak centered around 100–150 nm, which is like the size range observed by SEM. The intensity distributions are highly sensitive to larger particles and aggregates since nanoparticles scatter light in proportion to the sixth power of their radius, which make them particularly useful to detect small amounts of larger particles or aggregates. Figure 2a shows additional peaks in the higher range, indicating larger aggregates; however, these results should be interpreted with caution since DLS in this range is generally unreliable. To overcome this limitation of intensity distribution, Figure 2b shows the volume distribution. The volume-weighed distributions show the dominant size ranges in the distribution, with multiple peaks between 10 and 100 nm. The volume distribution does not indicate peaks at larger sizes, showing that the contribution of larger aggregates is negligible in the system and that the smaller particle fractions are the main contributors to the overall particle volume.^52^

**Figure 2:**
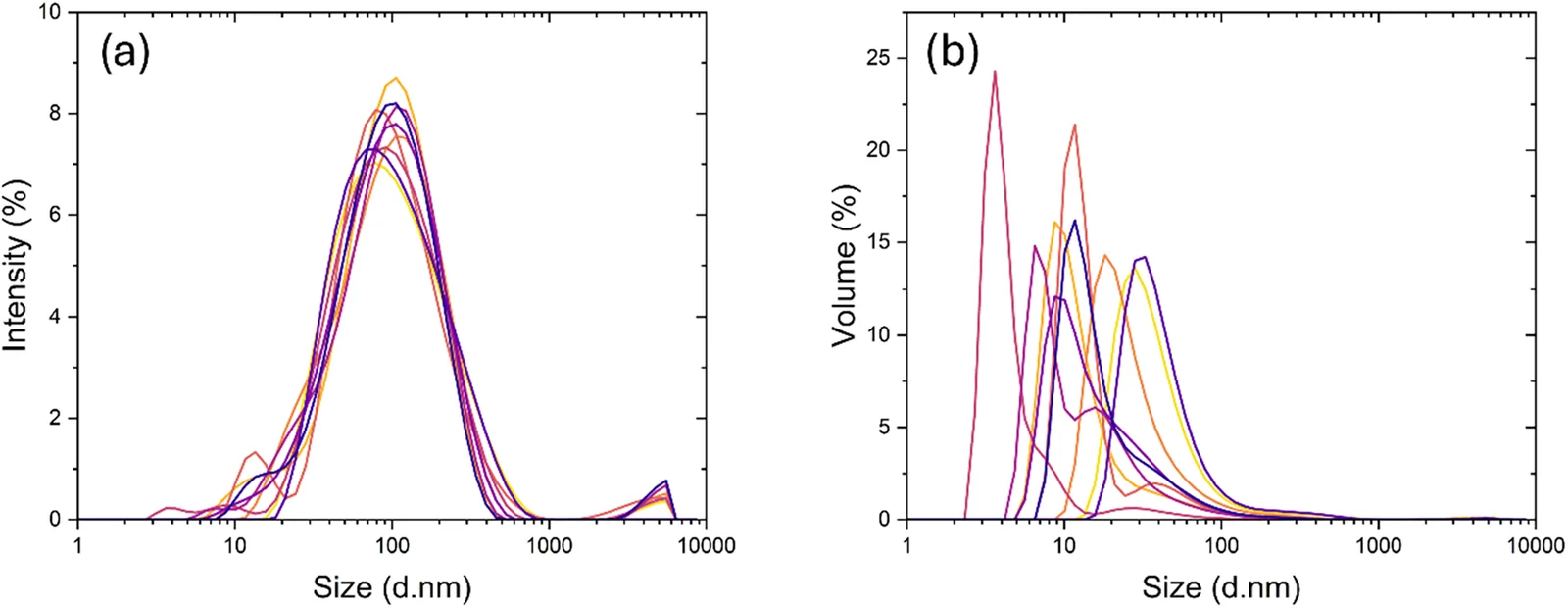
DLS distribution of BLGNP (1 wt %) dispersed in distilled water. The size here is the diameter (*d*). The multiple curves represent repeated measurements. (a) Intensity-weighted size distribution and (b) volume-weighted size distribution.

We also studied the zeta potential of BLGNPs at four salt concentrations (0, 5, 10, and 20 mM NaCl) and three particle concentrations (1, 3, and 5 wt %); see Figure 3 for an overview. At 0 mM NaCl, the zeta potential decreases from −46.5 mV at 1 wt % to −39 mV at 3 and 5 wt % particle concentrations. Thus, as particle concentration increases, the apparent surface charge density decreases due to particle–particle interactions.^53^ Upon the addition of 5 mM salt, the zeta potential became less negative from −32.1 mV at 1 wt % to −28.6 mV at 3 and 5 wt % particle concentrations. The reduction in the zeta potential with addition of salt is attributed to electrostatic screening by NaCl ions. Further, the observed decrease in potential with increase in particle concentration is likely due to a tendency for particle aggregation.^54^ In 10 and 20 mM NaCl, the zeta potential values continued to be reduced with the increasing particle concentration. Overall, the results indicate that zeta potential values of BLGNPs are strongly influenced by particles and ionic strength. This aligns with electrostatic stability and the reduction of Debye length and the subsequent reduction of zeta potential. Our studies indicate that negative zeta potential in the range of −30 mV generally gives reasonable colloidal stability. Stronger negative zeta potential does not necessarily enhance stability, as emulsions without salt in the aqueous phase tend to undergo phase separation much quicker than emulsions that have salt in the aqueous phase. This could be due to excessive interparticle repulsion between the particles, whereas a moderate particle aggregation induced by salt and higher particle concentrations contributes to improved emulsion stability.

**Figure 3:**
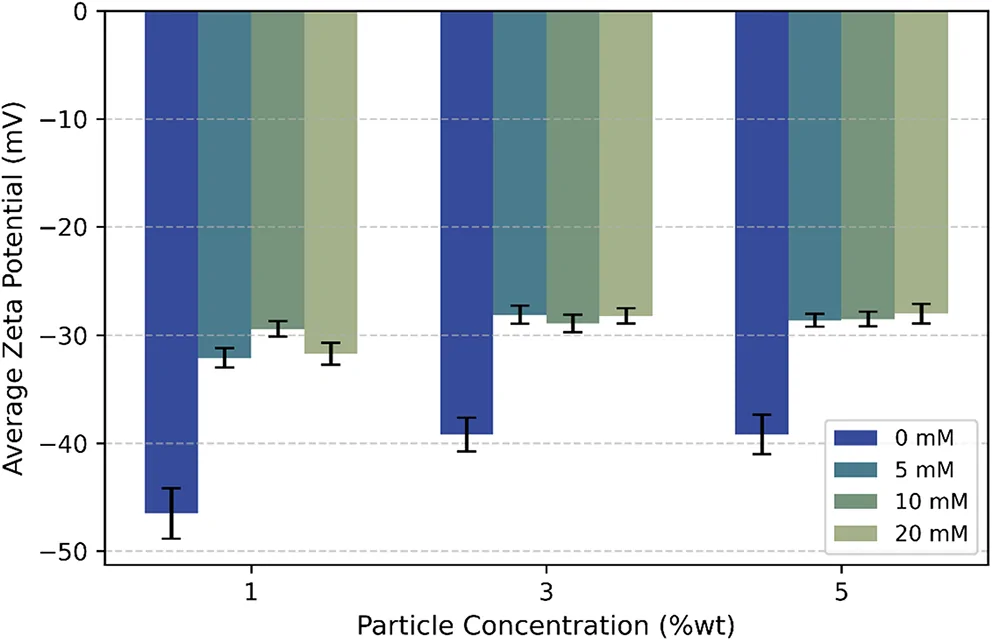
Zeta potential of BLGNP dispersions for different particles and salt concentration in the aqueous phase.

### 3.3 Physical Storage Stability of the Emulsions

Figure 4 shows a panel of emulsions prepared with 75 wt % of dispersed-phase oil fractions, stabilized with varying BLG nanoparticle concentrations. The emulsions were stored at room temperature and monitored visually for the assessment of physical stability. They were observed over multiple days, and images show changes, like creaming and phase separation.^55,56^ The emulsions containing 2 wt % particle concentrations (Figure 4, top row) exhibit a moderate to significant degree of creaming over time, with a dense, opaque upper layer and water-rich lower layer. Meanwhile, emulsions prepared with higher particle concentrations (3 and 4 wt %) exhibit better homogeneity and more uniform appearance, with no visible phase separations. The texture remains dense and homogeneous, indicating a well-packed structure for stable HIPEs.^57^

**Figure 4:**
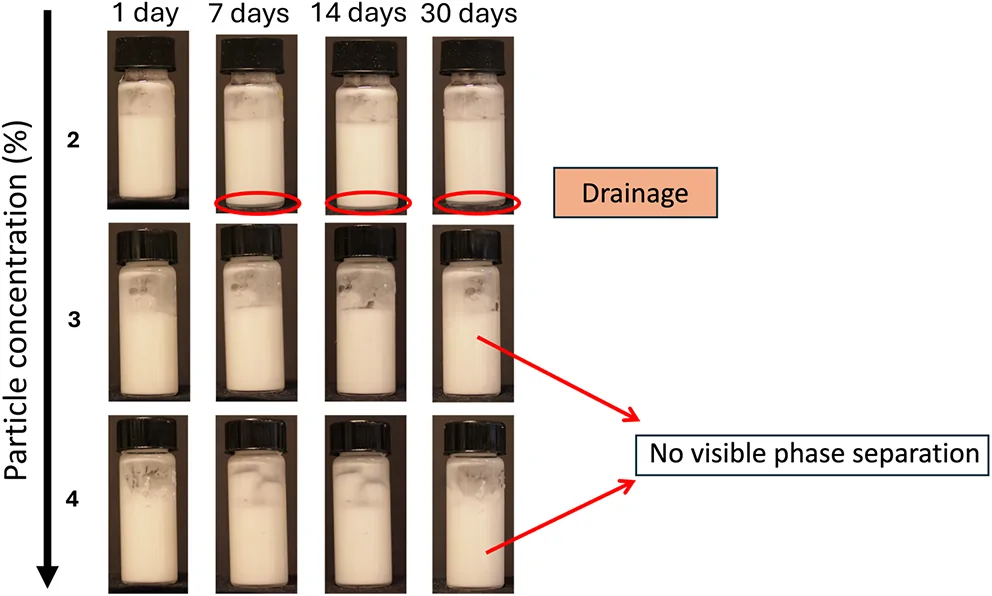
Visual stability of high-internal-phase BLGNP-stabilized emulsions (75 wt % oil) with varying particle concentrations observed over time. Samples were photographed on Day 1, Day 7, Day 14, and Day 30. Rows correspond to increasing particle concentrations (top to bottom) while columns represent the time points (left to right).

These visual results are useful as macroscopic tests and underline the role of particle and oil concentrations in stabilizing the HIPE. Previous studies have demonstrated that HIPEs are more stable and exhibit solid-like behavior due to high oil concentrations.^58^ At lower particle concentration (2 wt %), insufficient particle coverage at the oil–water interface likely led to partial coalescence and creaming, resulting in phase separation.^17^ With increasing concentration of BLGNPs, stabilization improves significantly. Nanoparticles irreversibly adsorb at the interface, forming a layer around the oil droplets.^59^ Furthermore, BLGNPs impart a mild surface charge (cf. ζ-potential), contributing to electrostatic repulsion, which further minimizes the aggregation. The emulsions with higher BLGNP concentrations remained kinetically stable even after prolonged storage, confirming that a threshold BLGNP concentration is necessary to suppress the phase separation and droplet migration.

### 3.4 Optical Microscopy on Emulsions

Figure 5 shows the optical microscopy images of Pickering HIPEs with increasing nanoparticle concentrations from 2 wt % to 5 wt % and dispersed phase of 75 wt %. The mean droplet diameter, estimated from the measurements made with imageJ software, was found to be in the range of 4–10 μm. Note that these values should be interpreted with caution since the droplets were measured manually and due to the ambiguities in identifying the droplets in the image. This can be seen particularly in the case of emulsion with lowest particle concentration (2 wt %). The droplets exhibit a wide size distribution with a large number of fuzzy, irregularly spaced droplets. There is no clear visual boundary for the droplets, and they appear diffuse and are often found merged, indicating coalescence. This could be because of instability due to incomplete coverage of the droplets by the Pickering particles. These emulsions tend to behave more like liquids, with droplets that are mobile. This suggests that 2 wt % BLGNPs is not adequate for completely covering the oil–water interface and preventing the phase separation of the emulsions.^17^

**Figure 5:**
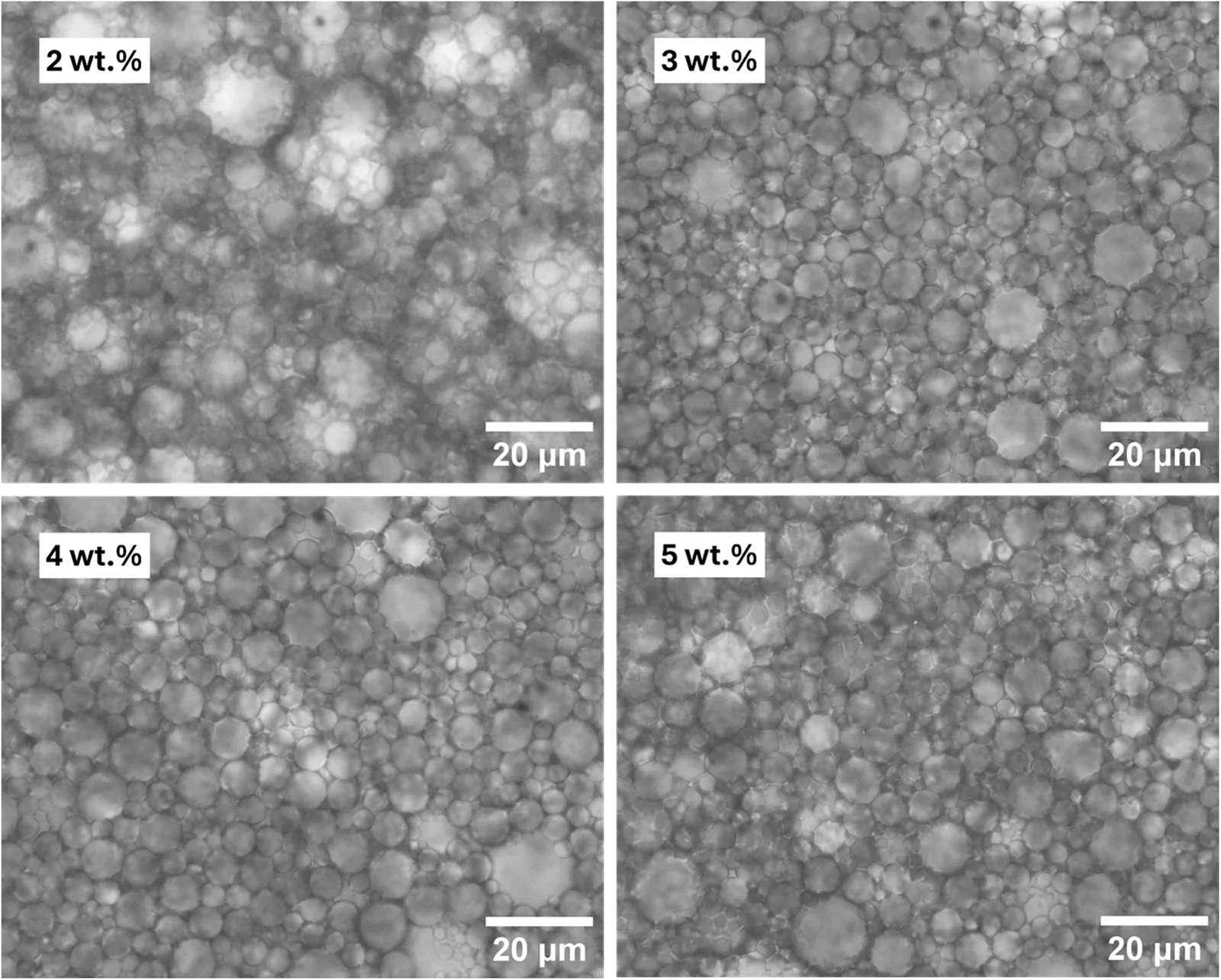
Optical microscopy images of high-internal-phase Pickering emulsions prepared with dispersed phase of 75 wt %. The micrographs show the effect of particle concentration on droplet size distribution. The scale bar is 20 μm.

With an increase to 3 wt %, a noticeable change in the emulsion structure could be observed. The droplets appear to be smaller with a more uniform distribution. When the particle concentration increased from 2 to 5 wt %, the packing density changed from loose and disordered packing to a tightly packed structure. At 3–5 wt %, the emulsions show generally spherical droplets, but some exhibit slightly polyhedral shape due to close packing. Optical microscopy images directly correlate with the visible stability of the emulsions (refer to Section 3.3). For the lower concentration, insufficient particle coverage leads to coalescence and an eventual phase separation. After a certain threshold of particle concentrations, the emulsions are observed to be more stable with a uniform distribution and no visual phase separation.^58,60–62^

Earlier studies conducted on cross-linked BLGNP emulsions with a dispersed phase of 75 wt % also showed similar results to what we observed in our emulsions.^17^ The network formation seen for higher concentrations contributes to the long-term shelf life stability of the emulsions.^63^

### 3.5 Rheological Behavior

The rheological properties of high-internal-phase Pickering HIPEs stabilized by BLGNPs were investigated using frequency sweep measurements as shown in Figure 6. The figure shows measurements on the HIPEs prepared by varying particle concentrations (2 and 5 wt % in Figure 6a), varying the salt concentrations in the aqueous phase (5 mM and 10 mM in Figure 6b) and the effects of aging (Figure 6c,d). Across all conditions, the storage modulus (*G*′) dominates over the loss modulus (*G*″) throughout the tested frequency range (0.01–100 rad/s), indicating a solid-like behavior, which is a common characteristic of HIPEs.^64^ The strong upturn for *G*′ at the highest oscillation frequencies reflects the time-dependent mechanical response of the microstructure and is most likely due to a combination of different effects that counteract deformation and thereby increase the apparent stiffness: (1) the droplets in the emulsion have no longer sufficient time to rearrange or flow past each other and (2) the dominating effect of the interfacial tension and hydrodynamic interactions between droplets at the highest frequencies.^65–67^ However, the absolute value of *G*″ is still relatively high (>100 Pa), as expected for HIPEs due to the high friction between adjacent layers of droplets that are in contact. The storage modulus of the emulsions is weakly dependent on frequency, especially at lower frequencies, suggesting the presence of a highly interconnected network structure formed by the nanoparticles at the oil–water interface.

**Figure 6:**
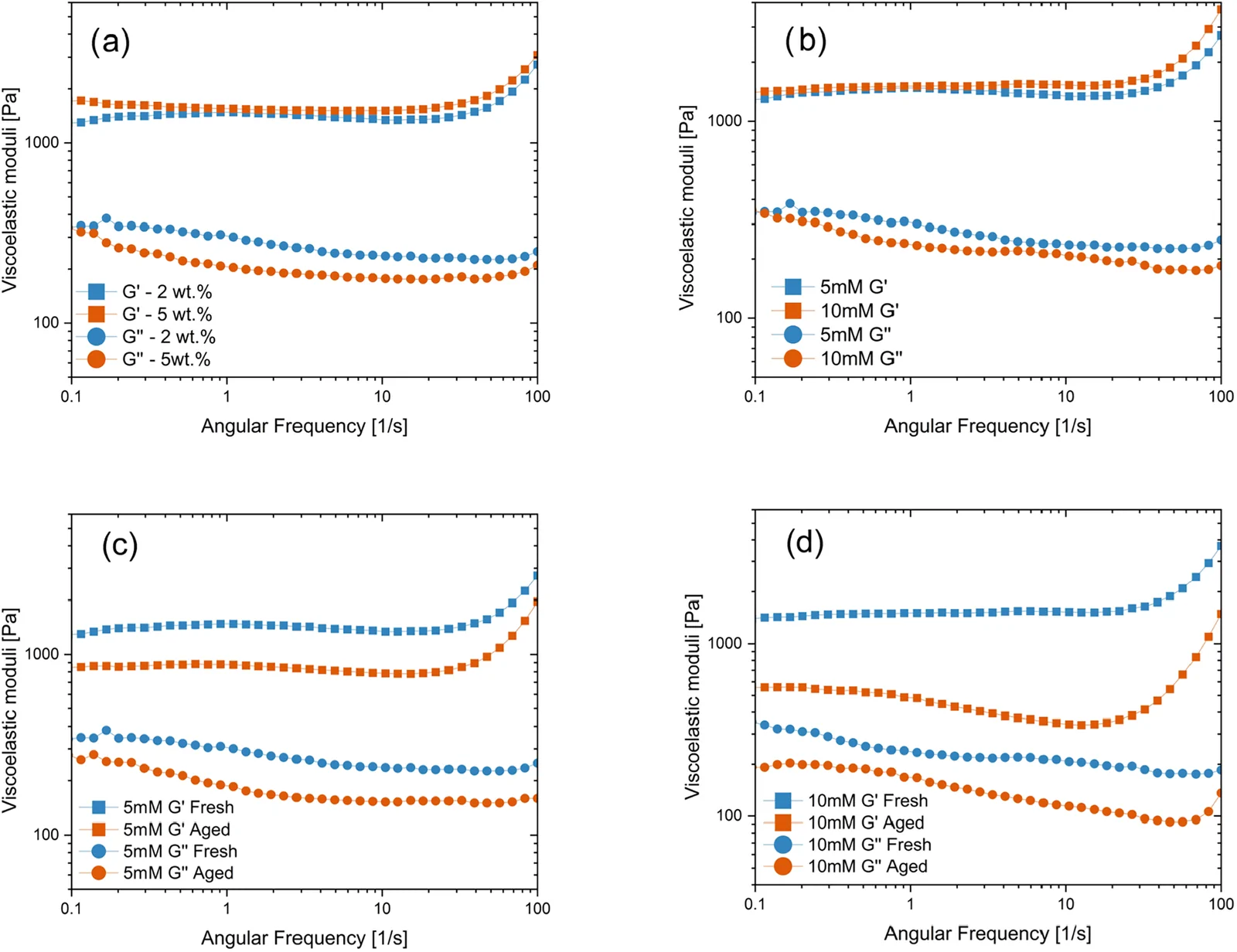
Frequency sweep measurements showing the storage modulus (*G*′) and loss modulus (*G*″) of Pickering high-internal-phase emulsions (HIPEs) under different conditions. (a) Effect of particle concentration (2 and 5 wt %) at fixed salt concentration in the aqueous phase (5 mM). (b) Effect of salt concentration in the aqueous phase (5 mM and 10 mM) at fixed particle concentration (2 wt %). (c) Comparison of fresh and aged emulsions (after aging) for 5 mM salt and 2 wt % particle concentration. (d) Comparison of fresh and aged emulsions for 10 mM salt and 2 wt % particle concentration.

Figure 6a demonstrates the effect of particle concentrations (2 and 5 wt %) at a fixed salt concentration of 5 mM. *G*′ is higher for 5 wt % across the entire frequency range, indicating that high-internal-phase Pickering emulsions are relatively stable, although some structural change is observed during shear for the highest concentration (5 wt %) of the droplets studied. Also, *G*′ shows only minimal frequency dependence (apart from the highest frequencies), suggesting a stable network. Experiments have shown that increasing particle concentration strengthens the HIPEs structure. Higher particle concentration ensures more complete coverage of droplet interfaces and promotes the formation of a denser interdroplet network. This strong network prevents the droplets from coalescing and prevents the phase separation of the emulsion.^68^

Figure 6b shows the effect of salt concentration (5 mM vs 10 mM) at a fixed particle concentration (2 wt %) on HIPEs. Here, emulsions were prepared by first dispersing particles in the aqueous phase containing 5 mM and 10 mM salt. The *G*′ values are stable and higher for 10 mM emulsions than 5 mM emulsions across most of the frequency range. This shows that the emulsions prepared with higher salt concentrations form a stronger mechanical network. At higher ionic strength, the nanoparticles are screened, allowing particles to pack more densely at the droplet interface. Closely packed particles enhance the interfacial rigidity and the droplet–droplet connectivity, leading to increased elasticity. Both 5 mM and 10 mM emulsions show weak frequency dependence of *G*′, which is typical for Pickering HIPEs. However, the 10 mM emulsions show an even flatter *G*′ curve, indicating stronger particle interactions and more “gel-like” behavior over time scales corresponding to low frequencies.^69^

If we compare with data found in Miao et al.^69^ on HIPEs stabilized with cellulose nanocrystals, our storage modulus *G*′ is in the range around 10^3^ Pa compared to 10^3^–10^4^ Pa for Miao et al.^70^ Also, the loss modulus is in the same range, around 3 × 10^2^ Pa compared to 10^3^ Pa. This similarity is interesting considering the very different particles for stabilization and the higher amount of oil used (80%). Figure 6c,d presents the effect of aging on the viscoelastic properties of HIPEs, under different salt conditions. Fresh and aged samples were also compared by measuring *G*′ and *G*″ across the frequency range. For 10 mM salt emulsions, aging leads to a substantial reduction (50–60%) in storage modulus, indicating a partial structural breakdown of the emulsions. Over time, HIPEs experience partial structural relaxation, possibly due to rearrangement of droplets, droplet coalescence, and Ostwald ripening. These phenomena reduce the number of effective droplet–droplet interactions and weaken the elastic network. Interestingly, emulsions with 5 mM salt show lower susceptibility to structural changes with aging compared to 10 mM salt emulsions. The 5 mM emulsions only show a smaller decrease (∼30%) in *G*′ after aging and minimal changes in frequency response. This behavior highlights the critical role of ionic strength in long-term mechanical stability of Pickering HIPEs, where particle packing at higher salt concentrations shows more resistance to aging.

It has been commented that for high stability, a network is needed also in the continuous phase.^71^ Therefore, the observed lowering of *G*′ for aged samples can reflect degradation of the interconnections within the aqueous phase, possibly due to long-term migration of particles from the water to the oil–water interface. However, in this respect, there may be large variations depending on particle type, size, charge, etc. It should also be noted that polydispersity in droplet size may influence HIPE rheology through its effect on droplet packing and ordering. This is normally not accounted for in rheology models, such as the Princen model or the Otsubo and Prud’homme analysis.^72^

With respect to the viscous properties, the steady shear viscosity shows clear shear-thinning behavior for all samples. This is exemplified in Figure 7, where there is a highly systematic trend with a log–log slope near −1.0 over approximately 5 decades in shear rate (see also the Supporting Information for additional rheological data and fitting results). The data was fitted to the Ostwald–de Waele empirical relation,^17^ η­(γ̇) = Cγ̇^*n*–1^, to obtain the proportionality coefficient *C* (consistency index) and the power law exponent (flow index −1), as shown in the inset box in Figure 7. Such strong shear thinning is expected due to the rearrangement and deformation of emulsion droplets in the plane of the applied shear force.^72^ The behavior resembles that of Miao et al. on CNC-stabilized emulsions, although these authors observed more variation with shear rate for the highest oil concentration (80%).^73^ We note a nearly complete overlap between the curves for the fresh and aged samples (same for 5 wt %; see Figure S4), indicating that the microstructure responsible for droplet and particle–particle interactions is largely maintained during aging, at least for the 5 mM system (Figure 7a). For higher amounts of salt, i.e., 10 mM (Figure 7b), the shear-thinning behavior is maintained, but there is a drop in the absolute viscosity (more than a factor of 2) for the aged sample, as seen also from the fitting parameters in the inset. Thus, the screening induced by the higher molarity probably induces (with time) aggregate/cluster formation and thereby a lower overall viscosity.

**Figure 7:**
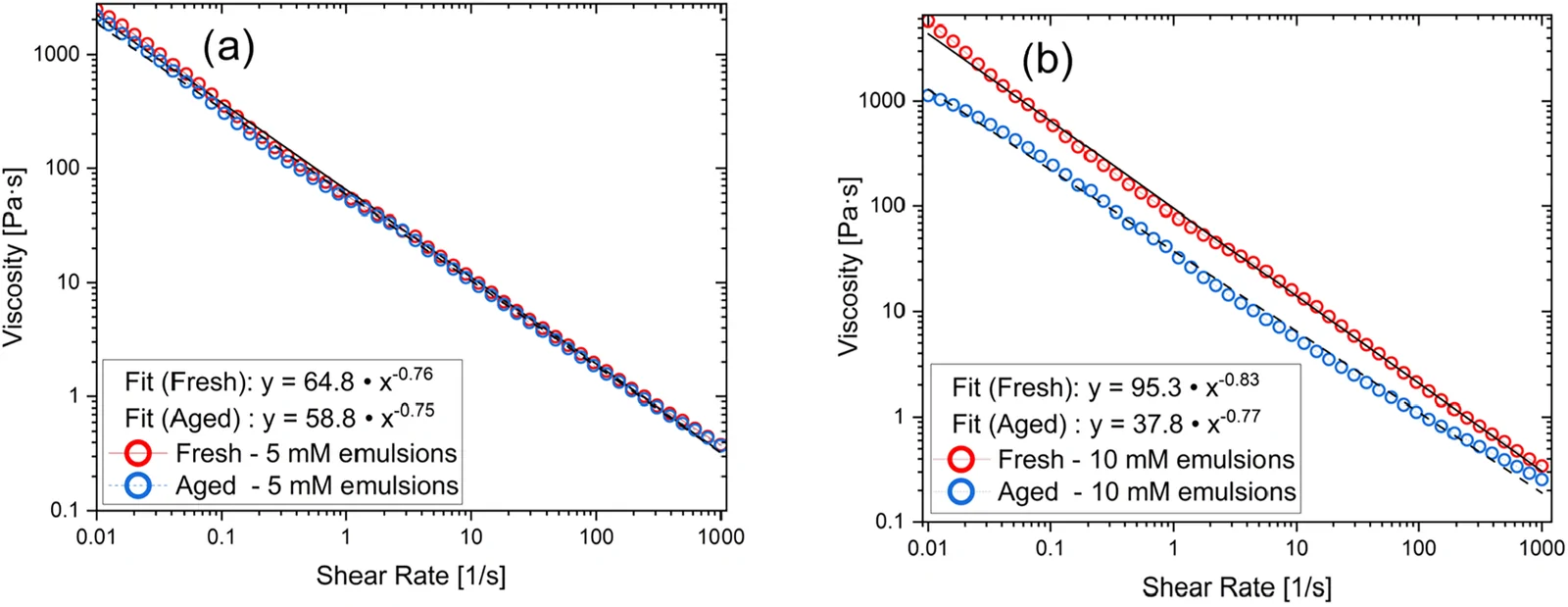
Steady shear viscosity as a function of shear rate for 2 wt % particle-stabilized emulsions with 5 mM (a) and 10 mM (b) salt concentration, comparing fresh and aged samples. The data have been plotted as log–log and was fitted to the Ostwald–de Waele relation (*y* = viscosity and *x* = shear rate), giving the power law slope (*n* – 1) and scaling coefficient (C) shown in the inset box.

Figure 8 shows the shear stress data for the same two samples as in Figure 7. From Figure 8, it is evident that the 5 mM system shows nearly identical behavior for the fresh and aged samples, with just a slight yield stress reduction for the aged samples, whereas for the 10 mM system, there seems to be a large reduction, although these data are noisier and the trends less clear. This difference with different salt concentrations is also in accordance with the observed viscosity behavior (Figure 7), underlining the observation that above a certain concentration of salt, the stability is reduced, most likely due to the increased tendency for particle clustering with stronger screening of the particle charges. The existence of such a screening effect is clear from the zeta potential (cf. Figure 3), at least 0–5 mM salt. On the other hand, there are just minimal zeta potential changes from 5 to 10 mM, and one cannot exclude that for these emulsions, other factors, e.g., entropic effects of ionic species in the aqueous phase, contribute to the observed change of stability with the higher salt concentration.

**Figure 8:**
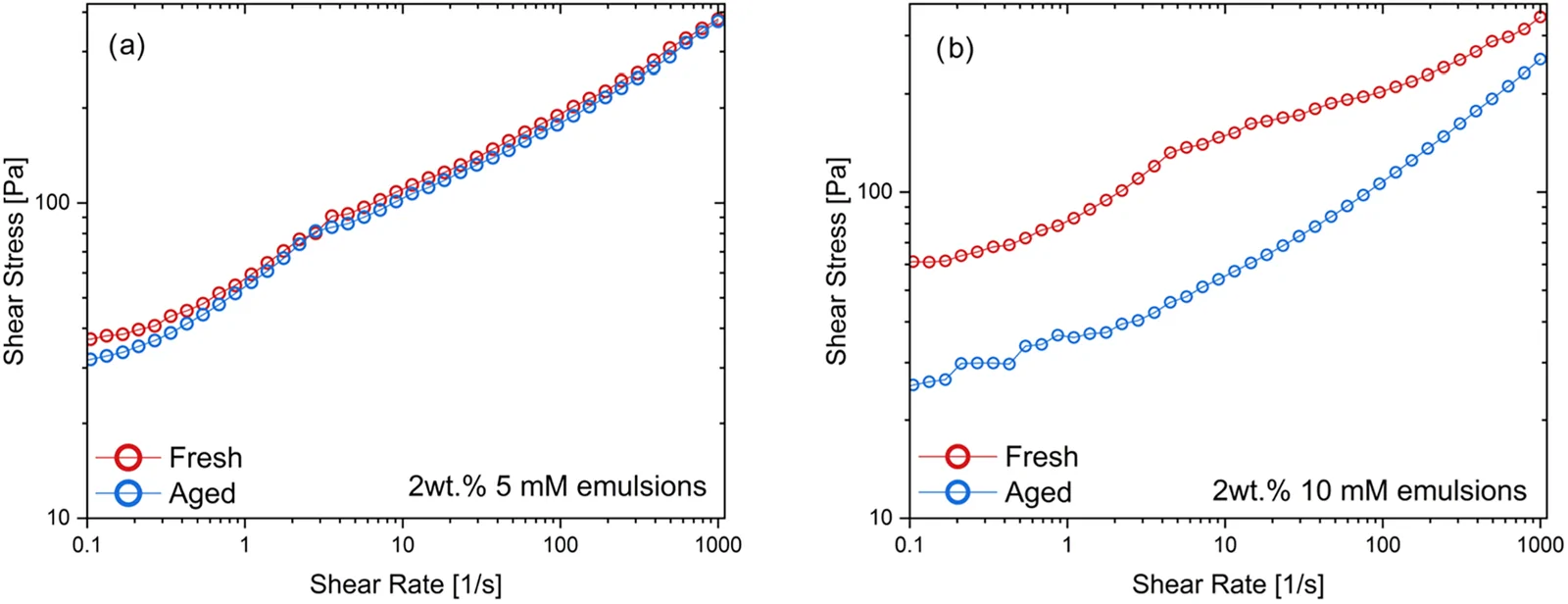
Shear stress as a function of shear rate for 2 wt % particle-stabilized emulsions with 5 mM (a) and 10 mM (b) salt concentration, comparing fresh and aged samples. The data have been plotted as log–log and was fitted to the Herschel–Bulkley model, giving the yield stress value (τ_*y*_), the consistency index *C*, and the flow index *n*.

### 3.6 Structural Characterization of Particles and Emulsions

#### 3.6.1 Effect of Concentration and Salt on Particle Dispersions

Before conducting X-ray experiments on the emulsions, BLGNP dispersions were investigated separately under different particle and salt concentrations. Dispersions of BLG proteins have been studied before but not BLG particle dispersions.^28,74,75^ For this purpose, BLGNPs were dispersed in different salt concentrations (5 mM and 10 mM NaCl solutions) at pH 9 (article dispersions in 20 mM NaCl solution, together with the corresponding fitting parameters, are presented in the Supporting Information) and measured at room temperature (25 °C). Representative scattering curves are shown in Figure 9, and the selected parameters from fits to the data are listed in Table 1.

**Figure 9:**
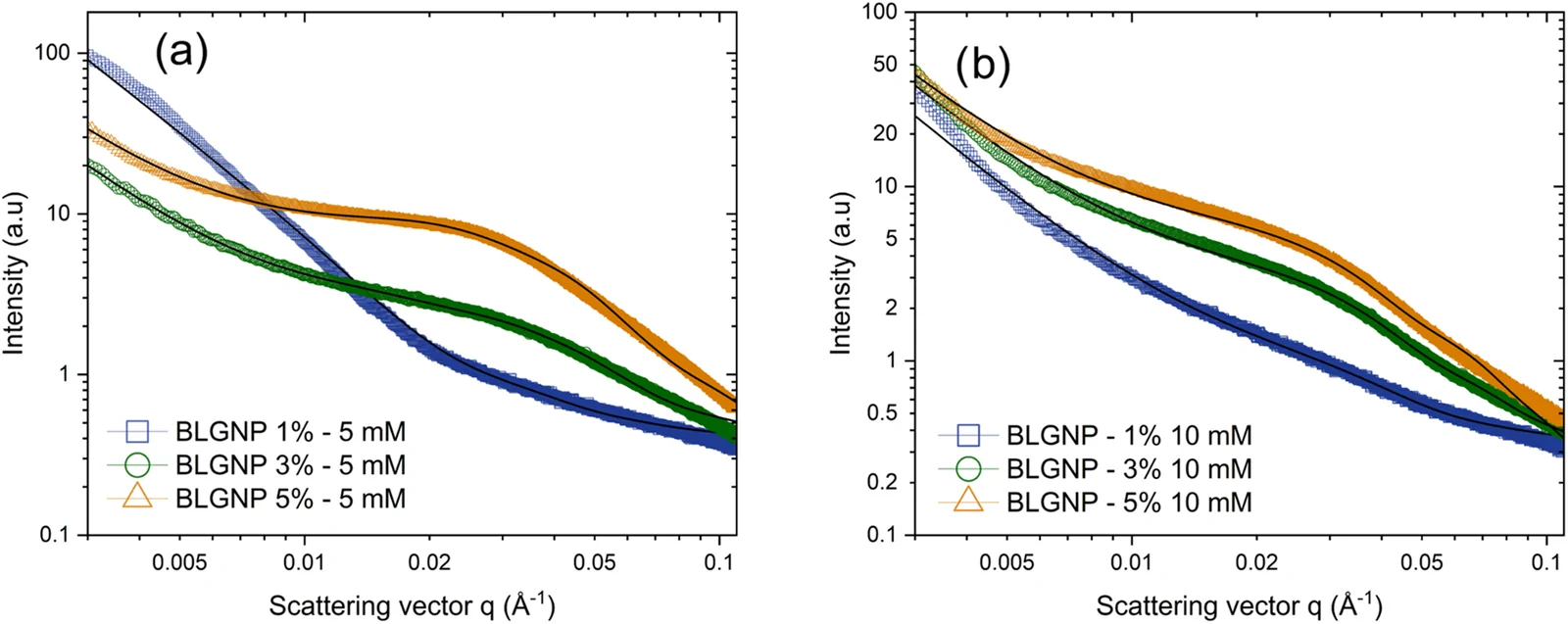
SAXS data for 1, 3, and 5 wt % nanoparticle dispersions in aqueous solution with (a) 5 mM and (b) 10 mM NaCl concentration, showing Intensity vs scattering vector *q* measured at room temperature (25 °C). The data were fitted using a Guinier–Porod model combined with an ellipsoidal model with Hayter–Penfold MSA as the structure factor. The colored symbols represent the experimental data and the black line indicates the model fits.

**Table 1:** Selected Structural Parameters Deduced from the Fits to the Data of Particle Dispersions with 1, 3, and 5 wt % Particles under Different Salt Concentrations (5 and 10 mM) Measured at Room Temperature (25 °C)^a^

sample name	*R*_p_ (Å)	*R*_eq_ (Å)
BLGNP/1/5	9.8 ± 1	122 ± 1
BLGNP/3/5	10.4 ± 1	59.2 ± 1
BLGNP/5/5	10.1 ± 1	62.3 ± 1
BLGNP/1/10	13.2 ± 1	76.0 ± 1
BLGNP/3/10	13.6 ± 1	88.0 ± 1
BLGNP/3/10	12.1 ± 1	95.3 ± 1

a Here *R*_p_ and *R*_eq_ are the average polar and equatorial radii of the BLGNP particles. Note that these errors are larger than the intrinsic standard deviations produced by SasView (< ±0.5 Å).

All the data were fitted using a combined scattering model of Guinier–Porod and an ellipsoid with Hayter–Penfold MSA as the structure factor. The dimensional parameter for the Guinier–Porod model was set as zero for fitting the data in the low-*q* region (below 0.01 Å^–1^). This reflects the scattering from the largest particles.^76^ The scattering in the mid/high-*q* region deviated from that of spherically shaped entities, and the elliptical model was found suitable to describe their anisotropy. According to Table 1, the overall extension of these particles (counted as 2 × *R*_eq_) is in the range of 120–240 Å (or 12–24 nm). This corresponds approximately with the center of the range of volume distributions extracted from DLS (Figure 2b), with the distinction that SAXS probes asymmetry in the particle shape (in contrast to DLS). Along with the elliptical model, the structure factor improves the model fit by including the interparticle interaction arising due to the relatively large concentrations of particles.^77–79^ The SAXS analysis thereby provides useful information regarding the shape, size, and interactions of the particles under different concentrations (1, 3, and 5 wt %) and salt concentrations (5, 10, and 20 mM) before being used in emulsions. The maximum size of the large particles, also present to some extent in the dispersion, cannot be accurately calculated from these data since there is no visible plateau in the lowest *q* range, and the data are relatively noisy in this region. Thus, the radius of gyration (*R_g_*) obtained corresponds to particles larger than 300 nm. This agrees with the results obtained from DLS, where indications of hydrodynamic diameters above 100 nm were also found.

A systematic increase in the scattering intensity is observed with the increase in particle concentrations. However, for 1 wt % (at 5 mM), the intensity in the low-q region was found to be higher than 3 and 5 wt %.^80^ In our previous work conducted on Pickering emulsions stabilized with BLGNP particles, we presented similar studies of BLGNPs dispersed in salt concentrations.^30^ In fact, while 3 and 5 wt % dispersions exhibited reproducibility across independent preparations, a notable distinction could be seen in the behavior of the particles dispersed at 1 wt %, for particles dispersed in 5 mM NaCl. This could be because at this concentration, the system is at the borderline of having particle clustering/aggregation, in contrast to the higher concentrations, where aggregation is more likely and reproducible (as observed from the systematic upturn in the lowest *q* range). This behavior also correlates with our observations that stable emulsions could not be made with 1 wt % concentrations, in contrast to 3 and 5 wt % systems.

With an increase in salt concentration, the polar radius and equatorial radius both increase. The fitting results thus suggest an average oblate elliptical shape (*R*_eq_ > *R_p_*) for particles in dispersion, in contrast to the cross-linked BLG nanoparticles discussed by Dai et al.,^17^ which took the form of prolate or elongated ellipsoids. The SAXS analysis confirms that salt and particle concentrations influence BLGNPs. However, the effect is quite small (at least for the part of the population that falls within the size range accessible with SAXS), indicating that particles prepared without cross-linking agents are also relatively stable structurally. These findings clarify the stability and structure of HIPE stabilized with BLGNPs, as discussed in subsequent sections.

#### 3.6.2 Effect of Particle Concentration and Salt on Emulsions

To investigate how particle and salt concentration affect the emulsion structure and stability, SAXS measurements were conducted for emulsions with varying particle concentrations (2–5 wt %) and ionic strength of the aqueous phase (0–20 mM). Emulsions were prepared by dispersing the protein nanoparticles in aqueous solutions of NaCl with different molarities. These samples were freshly prepared (Fresh HI-BLGNPem) and measured immediately after homogenizing the emulsions. Figure 10 shows the SAXS data of emulsions with varying particle and salt concentrations. It is to be noted that for 20 mM salt concentrations, emulsions were prepared for three particle concentrations (3–5 wt %) since the 2 wt % emulsions were destabilized immediately, undergoing aggregation and phase separation after preparation. The SAXS data were modeled using a hybrid plugin model feature available in SasView, combining a core–shell sphere model for the low-q region (0.003–0.01 Å^–1^) and an ellipsoidal model with Hayter MSA as the structure factor for the high-*q* region (0.01–0.1 Å^–1^). This helps capture the scattering from the oil-droplet interfaces and the continuous phase. The observed strong low-*q* scattering, with a power-law slope close to −4 in the lowest *q* range, is expected due to the oil droplets. This stems from the fact that the droplets are much larger than the size range accessible by SAXS (around 500 Å). The radius of the oil droplets was therefore fixed to a large value while fitting (1 μm) to satisfy the −4 slope in the low-*q* region. To avoid oscillations in the model fitting, polydispersity was used in the model fitting for the droplet radius and shell thickness. The fitted SAXS curves align well with experimental data, thus capturing the main characteristics of the sample.

**Figure 10:**
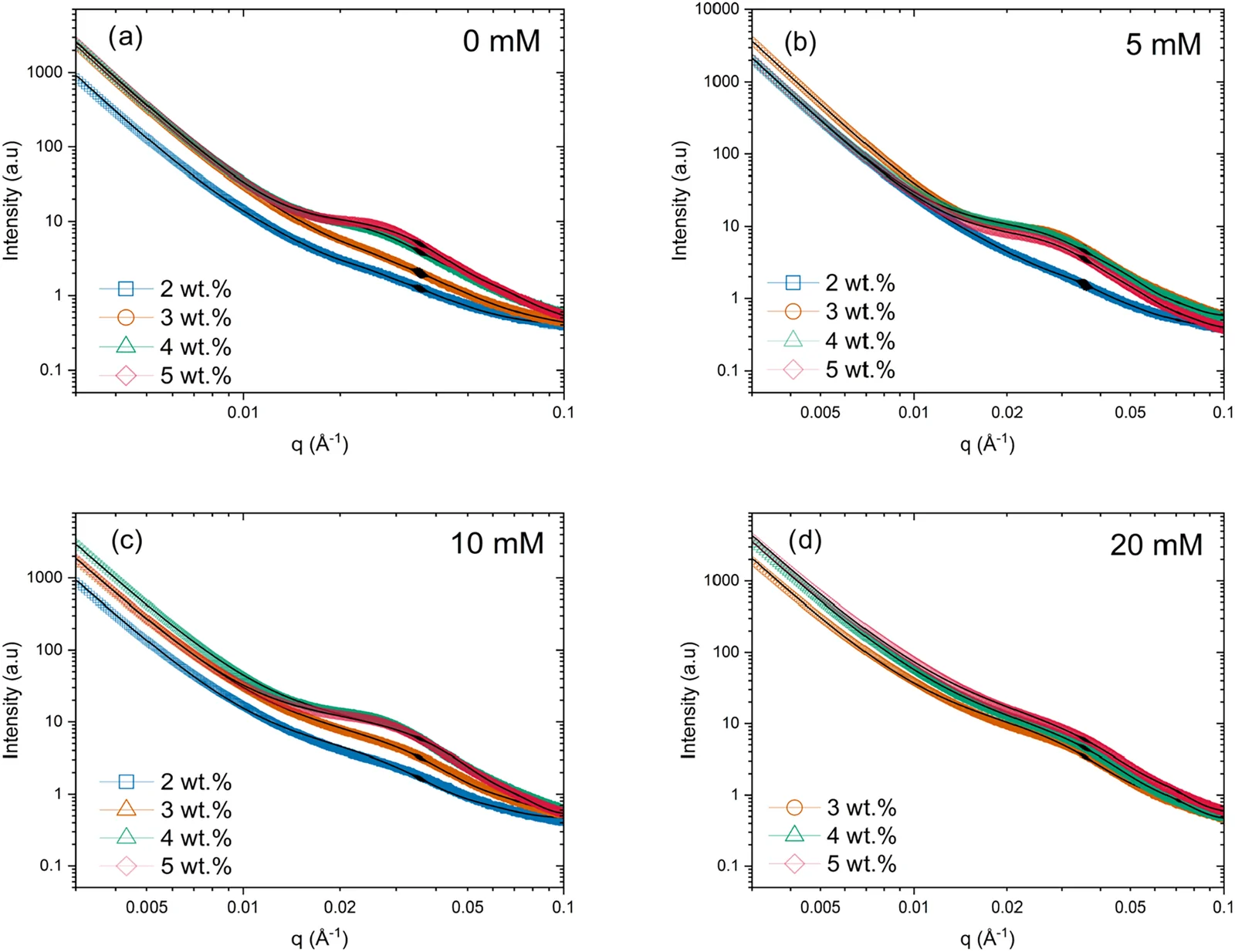
SAXS patterns of high-internal-phase Pickering emulsions stabilized with β-lactoglobulin nanoparticles (HI-BLGNPem) under varying particle and molar concentrations. The colored lines represent experimental data, while the black lines show the model fits. (a) HI-BLGNPem with 2, 3, 4, and 5 wt % prepared in a 0 mM continuous phase; (b) HI-BLGNPem with 2, 3, 4, and 5 wt % in 5 mM continuous phase; (c) HI-BLGNPem with 2, 3, 4, and 5 wt % in 10 mM continuous phase; and (d) HI-BLGNPem with 2, 3, 4, and 5 wt % in 20 mM continuous phase.

With an increase in protein concentration, stronger scattering is observed, especially at mid/high *q*. The “bump” observed at higher *q* values (0.03 Å^–1^) is fixed in position and becomes stronger with an increase in particle concentration for different amounts of salt. At 0 mM NaCl (Figure 10a), a clear dependence on concentration can be observed. Higher particle concentrations will generally enhance the interfacial coverage and may induce changes in droplet packing and deformation. For 2 and 3 wt %, the “bump” is merely visible, whereas for 4 and 5 wt %, it becomes pronounced, reflecting the stronger interparticle interaction at the interface and possibly also in the region between the droplets.^40^ For emulsions prepared with 5 and 10 mM salt in the aqueous phase (Figure 10b,c), the mid/high-*q* scattering features become more prominent also for 3 wt %, indicating that clustering due to increased screening is an important factor here. For emulsions with 20 mM salt concentrations, all curves coincide into a nearly identical profile regardless of the change in particle concentration. This suggests a saturation in the interfacial packing and coverage.^81^

From the SAXS data analysis, particle size parameters (equatorial and polar radii) could be obtained via the ellipsoidal model, and shell thickness (droplet interface layer thickness) was obtained from the core–shell sphere model. For 0 mM emulsions at 2 wt %, the equatorial radius is relatively large, indicating a more open structure. Increasing the concentration leads to a significant decrease in anisotropy, as the equatorial size decreases (cf. Table S3).

#### 3.6.3 Effect of Aging and Temperature on Emulsions

SAXS measurements were also conducted to study the effect of aging and temperature on emulsions using a fixed amount of salt; cf. Figure 11. Emulsions were prepared by dispersing particles in a 5 mM NaCl solution and homogenizing with sunflower oil with 0.75 wt %. The emulsions were studied at varying particle concentrations from 2 to 5 wt %. Aged samples were measured after the emulsions were stored at room temperature for 90 days. If the emulsions were less stable, they underwent phase separation (see Figure 4). The emulsions were analyzed as before using a model that combines a core–shell sphere model and an elliptical model with Hayter–Penfold MSA as the structure factor. The structural parameters obtained from the model fitting are shown in Table 2.

**Figure 11:**
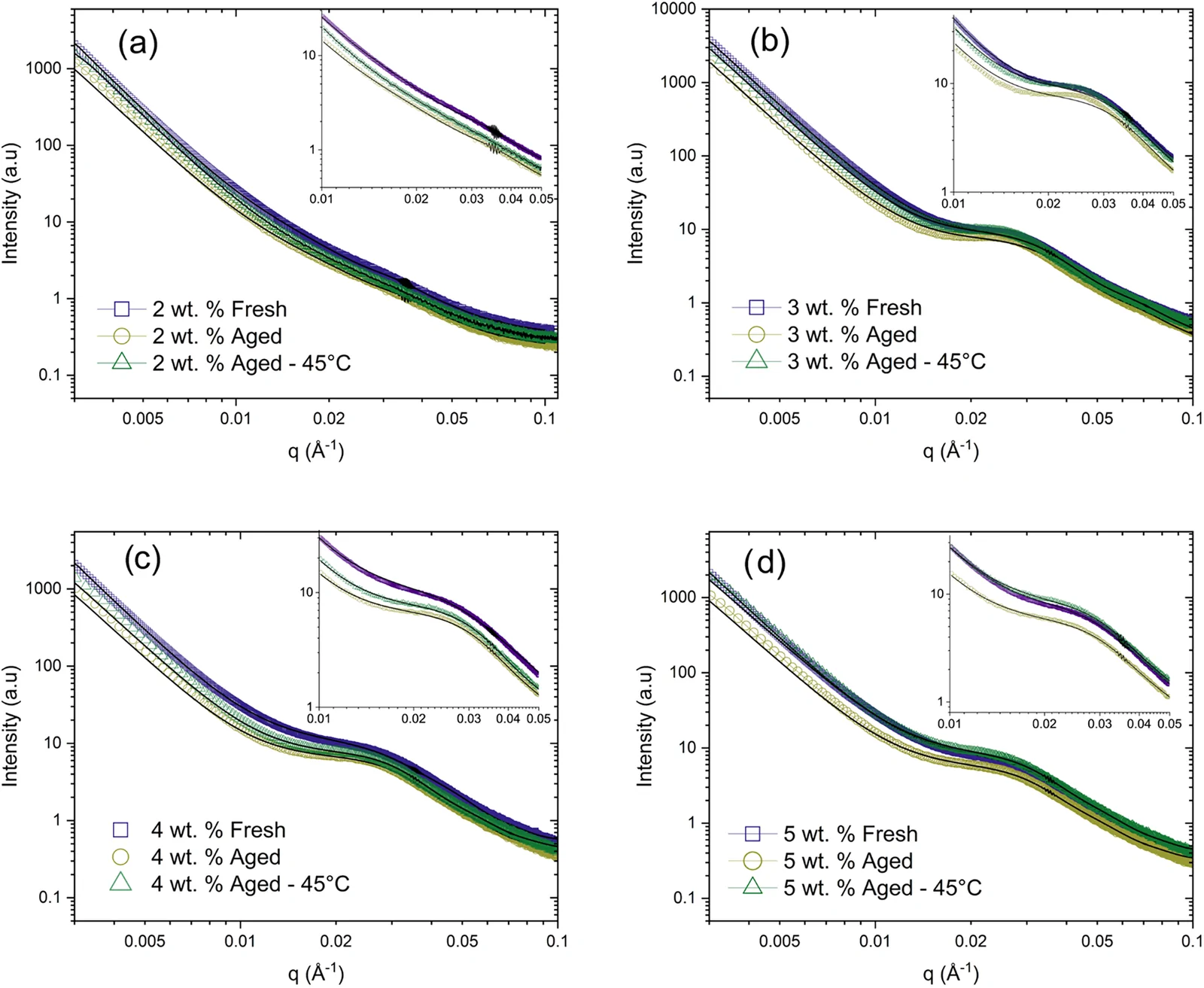
SAXS patterns of β-lactoglobulin nanoparticles (BLGNP) stabilized high-internal-phase Pickering emulsions with 75 wt % dispersed phases under different particle concentrations and three different measurement conditions (fresh, aged, and aged-45 °C). The inset figure highlights the mid-to-high-*q* region: (a) 2 wt %, (b) 3 wt %, (c) 4 wt %, and (d) 5 wt %.

**Table 2:** Model Parameters for BLG Particles in Emulsions with 2–5 wt % Particles in a 5 mM Aqueous Solution Prepared with 75 wt % Dispersed Phases as well as after Aging and Exposure to High Temperature^a^

sample name	*R*_p_ (Å)	*R*_eq_ (Å)	shell thickness (Å)
BLGNPem/2/5/75/fresh	34.0 ± 1	129.2 ± 1	102.0 ± 1
BLGNPem/2/5/75/aged	35.0 ± 1	74.3 ± 1	105.0 ± 1
BLGNPem/2/5/75/aged_HT	43.0 ± 1	58.2 ± 1	104.3 ± 1
BLGNPem/3/5/75/fresh	35.1 ± 1	73.2 ± 1	80.1 ± 1
BLGNPem/3/5/75/aged	41.0 ± 1	80.0 ± 1	66.3 ± 1
BLGNPem/3/5/75/aged_HT	39.0 ± 1	81.0 ± 1	75.0 ± 1
BLGNPem/4/5/75/fresh	22.0 ± 1	87.4 ± 1	97.0 ± 1
BLGNPem/4/5/75/aged	29.2 ± 1	89.0 ± 1	74.2 ± 1
BLGNPem/4/5/75/aged_HT	31.0 ± 1	91.0 ± 1	67.0 ± 1
BLGNPem/5/5/75/fresh	20.0 ± 1	79.0 ± 1	92.1 ± 1
BLGNPem/5/5/75/aged	20.0 ± 1	80.0 ± 1	84.4 ± 1
BLGNPem/5/5/75/aged_HT	27.0 ± 1	80.0 ± 1	84.0 ± 1

a Here, *R_p_* and *R*_eq_ refer to polar and equatorial radii of Pickering particles, and the shell thickness defines the interfacial layer of oil droplets. The model particle charge was 7.7 e^–^, salt concentration was 0.005, and dielectric constant of the dispersion medium was 71. The errors listed were calculated through a variation analysis using different starting values for the model parameters. Note that these errors are larger than the intrinsic standard deviations produced by SasView (< ±0.5 Å).

The model fitting shows that the polar radius is smaller than the equatorial radius for all samples, suggesting the average shape of an oblate ellipsoid, as for the particles in dispersion. The equatorial radius is found to be in the range of 73–89 Å (with slight increase upon aging, except for the 2 wt % samples that exhibit high *R*_eq_ for the freshly prepared samples). For the latter, a sharp decrease in radius is observed with aging. This could be because at this concentration, which is at the limit of stability, the system is more susceptible to changes in structure or reorganization of the particles at the interface. The shell thickness that corresponds to the size of the interfacial layer of the emulsions is close to 100 Å for fresh samples (except 3 wt %). The samples made with high-volume fractions are relatively uniform and do not undergo phase separation with time except for the low particle concentration (see Figure 4). The samples with 2 wt % show a slight increase in shell thickness with aging, suggesting coalescence and larger surface area of the oil droplets. With increasing particle concentrations (3–5 wt %), the emulsions do not undergo visible creaming or phase separation. For these samples, the shell thickness was found to decrease slightly with aging. This could be because of gradual (slow) loss of particles to the aqueous phase. All fitted SAXS curves could be well fitted with the model used here, and the results show that particle concentration significantly influences the interface structure and stability of the BLGNP-stabilized high-internal-phase Pickering emulsions during aging.

#### 3.6.4 RheoSAXS

RheoSAXS measurements were conducted on high-internal-phase Pickering emulsions stabilized by BLGNPs at two different concentrations (2 and 5 wt %) at a salt concentration of 5 mM in aqueous phase. The measurements were taken before shear and during maximum shear (1000 s^–1^). The inset graph illustrates the variations in the scattering profiles prior to and during shear by providing a zoomed-in view of the q region (0.01–0.05 Å^–1^). The intensity declines as a power-law decay, indicating that the scattering originates from emulsion structures, primarily the oil droplets and the interfaces between the droplets. Figure 12a shows the scattering intensity of emulsions with 2 wt % particles. During maximum shear, the intensity profile decreases slightly throughout the entire q range compared to the situation before shear, indicating a slight disruption of the internal structure.

**Figure 12:**
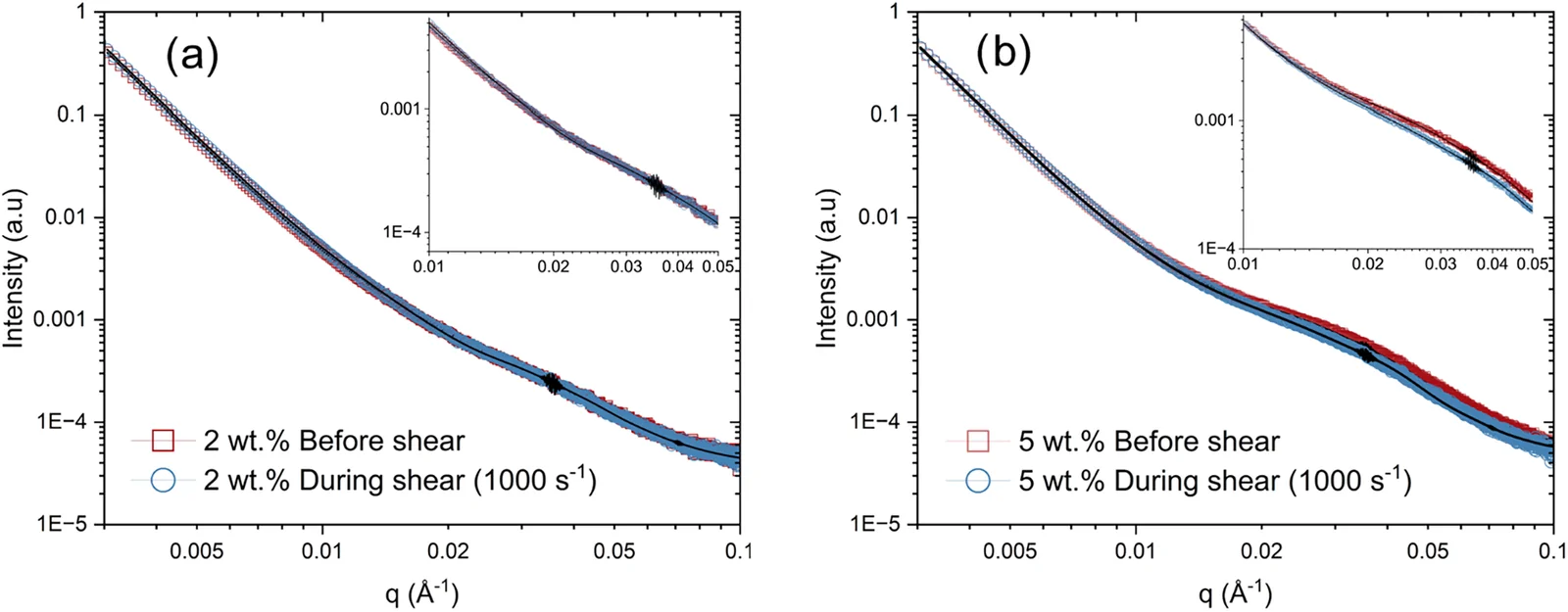
RheoSAXS figures of high-internal-phase Pickering emulsions before and during the shear. The inset figure highlights the mid-to-high-q region: (a) 2 wt % and (b) 5 wt %.

Figure 12b presents the scattering intensity for the emulsions with 5 wt % nanoparticles. The overall scattering profile is similar to 2 wt % but with higher intensity. This reflects a denser and more interconnected nanoparticle network due to the high concentration of the stabilizing particles. During shear, the SAXS profile shows a more pronounced decrease in intensity compared to the emulsion with lower concentration (2 wt %). This implies that while a higher particle concentration improves the emulsion stability, the internal structure is significantly disrupted by the shear force. After shear, the SAXS profile did not return to the original shape, showing that there is some irreversible structural change in the shear range probed.

Rheology measurements together with SAXS reveal useful information about the structural stability of high-internal-phase Pickering emulsions under shear. In general, the samples undergo only minimal structural changes before and during the shear, at least as judged from SAXS. This indicates that these high-internal-phase Pickering emulsions are relatively stable, although some structural change is observed during shear for the highest concentration studied (5 wt %). The emulsions retain their structure and stability to a significant extent, which makes them ideal for a food-grade system.

## 4 Conclusions

Here we explored the structural variations and rheological properties of Pickering HIPEs stabilized with BLG nanoparticles, using a combination of techniques such as visual observation, optical microscopy, scanning electron microscopy, rheology, and SAXS. SAXS was also combined with rheology (RheoSAXS) to follow the structural changes under deformation. HIPEs were prepared with high oil concentration (75%) and varying particle and salt concentrations, resembling food systems, and have previously not been investigated in this manner, especially considering SAXS on the effect of aging and rheology. The particles were prepared using an antisolvent technique without external cross-linking agents. The experiments showed good stability for concentrations of BLG nanoparticles above 3 wt %, and comparison between fresh and aged samples showed minimal differences, demonstrating structural stability that is advantageous especially for food-related systems. Along with aging, these samples were also observed at a high temperature and the results remained consistent. Thus, aging and temperature (up to 45 °C) did not alter the structure of the emulsions to a large degree.

While these emulsions were prepared by dispersing nanoparticles in the aqueous phase with different salt concentrations (0–20 mM), there is a limitation in preparing these emulsions with very high salt concentrations. This is because the particles undergo aggregation and clustering in the presence of the salt. Also, long-term storage of the particles (especially at higher salt concentrations) may lead to enhanced aggregation, compromising the stability of the prepared emulsions. In summary, the present work shows that HIPE prepared with BLG nanoparticles without any external cross-linking agent can produce stable food-grade emulsions, provided the BLGNP concentration is higher than 2 wt %.

## Supplementary Material



## Notes

Visual stability of emulsions with 5 wt % particle concentration observed over time; effect of particle concentration (2 wt % and 5 wt %) at fixed salt concentration (5 mM); zeta potential measurements were conducted on BLGNPs with different salt and particle concentrations; and selected structural parameters deduced from the fits to the data for BLG particle dispersions (PDF)

## ∥

Department of Physics, Norwegian University of Science and Technology, Høgskoleringen 5, 7034 Trondheim, Norway

## Abbreviations

BLG: β-lactoglobulin
BLGNP: β-lactoglobulin nanoparticles
BLGNP: solutions-β-lactoglobulin nanoparticles solutions
HIPE: high-internal-phase Pickering emulsions
BLGNPem: β-lactoglobulin nanoparticles stabilized emulsions
Fresh HI-BLGNPem: freshly prepared β-lactoglobulin nanoparticles stabilized high-internal-phase Pickering emulsions
Aged HI-BLGNPem: aged β-lactoglobulin nanoparticles stabilized high-internal-phase Pickering emulsions
Aged HI-BLGNPem HT: aged β-lactoglobulin nanoparticles stabilized high-internal-phase Pickering emulsions measured at 45 °C
