# Supplementary material for: High-Internal-Phase Pickering Emulsions Stabilized by β‑Lactoglobulin Nanoparticles: Effects of Rheology, Stability, and Aging
Source: Langmuir. 2026 Oct 22;42(43):31866–81. doi: 10.1021/acs.langmuir.6c04106 (PMC13738694; doi:10.1021/acs.langmuir.6c04106)
Supplement: Supplementary file 1 [file la6c04106_si_001.pdf]

Supporting Information

High internal phase Pickering emulsions stabilized by β-lactoglobulin nanoparticles: Effects of rheology, stability and ageing

Rini P. Ravindranathan*[1,2], Geir Helgesen[1], Matti Knaapila[2], Ann E. Terry[3], Jon Otto Fossum[2], Kenneth Dahl Knudsen[1,2]

[1] *Institute for Energy Technology, Instituttveien 18, 2007 Kjeller, Norway*

[2] *Norwegian University of Science and Technology, Høgskoleringen 1, 7034 Trondheim, Norway*

[3] *MAX IV Laboratory, Fotongatan 2, 224 84 Lund, Sweden*

Number of pages: 8

Number of figures: 5

Number of schemes: 0

Number of tables: 3

Table of Contents

1. Physical storage stability of the emulsions S2
2. Zeta potential S2
3. Rheological behaviour S3

4. Small-angle X-ray scattering S5
a. BLG particle dispersions S5
b. High-internal-phase Pickering emulsions S6

1. Physical storage stability of the emulsions

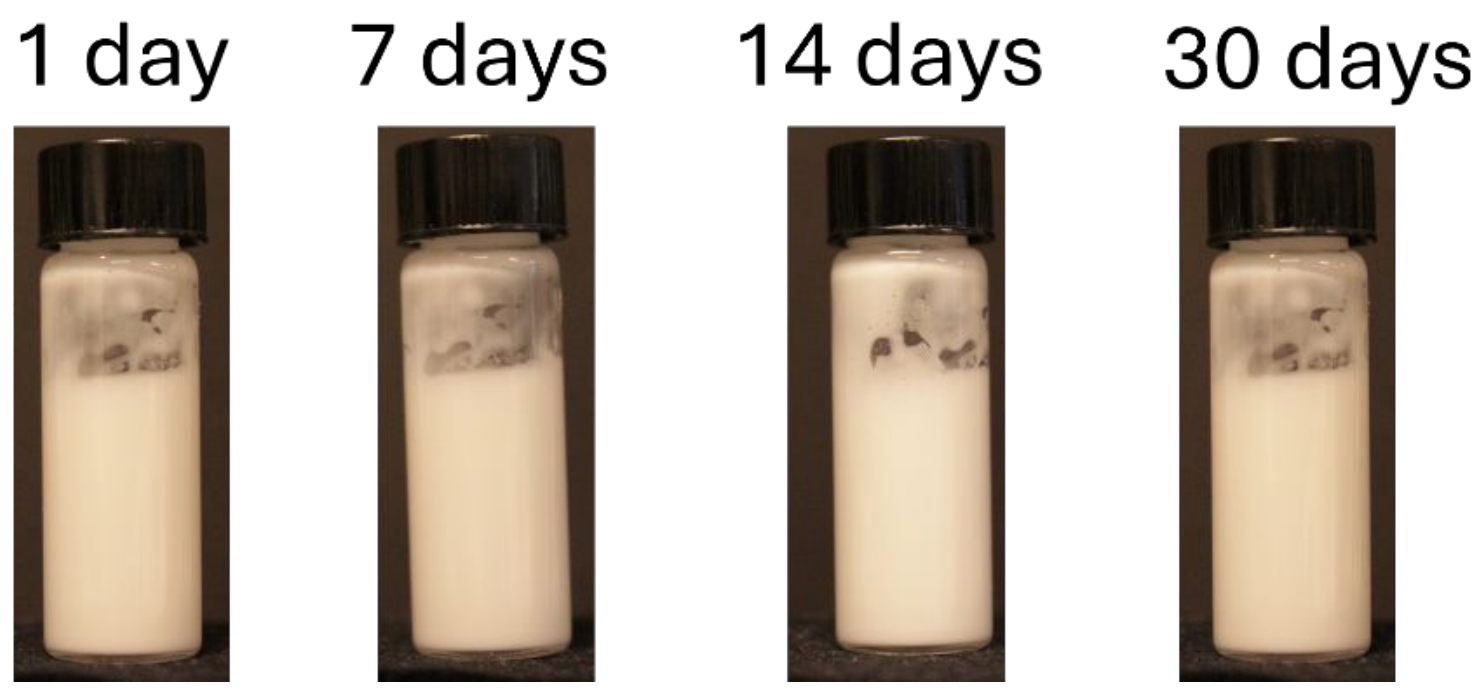

Figure S1: Visual stability of emulsions with 5 wt.% particle concentration observed over time. Samples were photographed on Day 1, Day 7, Day 14, and Day 30

2. Zeta potential

Table S1: Zeta potential measurements were conducted on BLGNPs with different salt and particle concentrations. All the solutions were at pH 9 with 0.02 wt.% of NaN3. An average of at least 10 measurements were taken

Sample	Particle concentration (%wt.)	Salt concentration (mM)	Average Zeta potential (mV)
BLGNP/1/0	1	0	-46.5 ± 2.34
BLGNP/3/0	3	0	-39.2 ± 1.56
BLGNP/5/0	5	0	-39.2 ± 1.83

BLGNP/1/5	1	5	-32.1 ± 0.895
BLGNP/3/5	3	5	-28.1 ± 0.837
BLGNP/5/5	5	5	-28.6 ± 0.606
BLGNP/1/10	1	10	-29.4 ± 0.72
BLGNP/3/10	3	10	-28.9 ± 0.808
BLGNP/5/10	5	10	-28.5 ± 0.677
BLGNP/1/20	1	20	-31.7 ± 1.01
BLGNP/3/20	3	20	-28.2 ±0.702
BLGNP/5/20	5	20	-28.0 ±0.909

3. Rheological behaviour

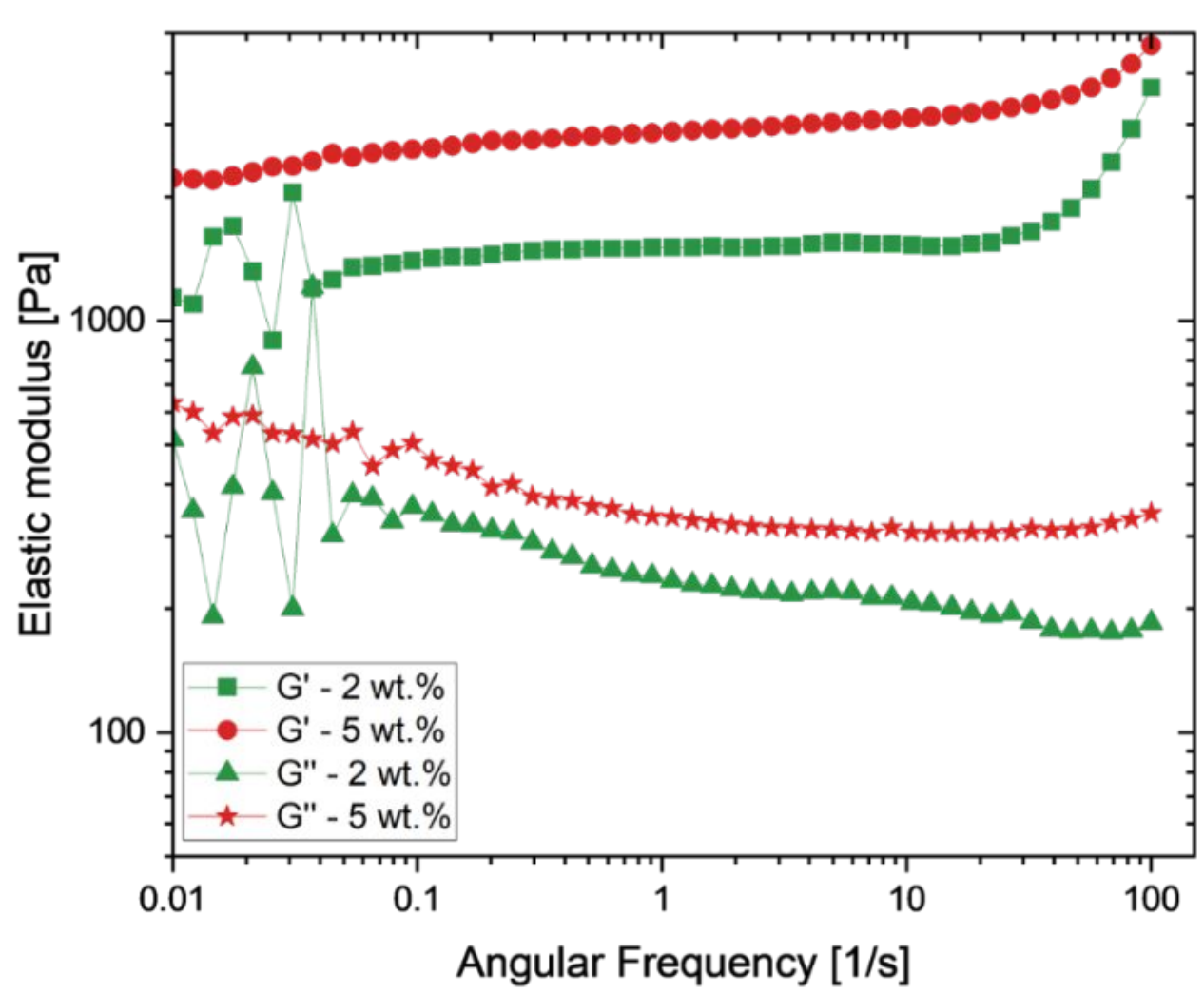

Figure S2: Effect of particle concentration (2 wt.% and 5 wt.%) at fixed salt concentration (5 mM).

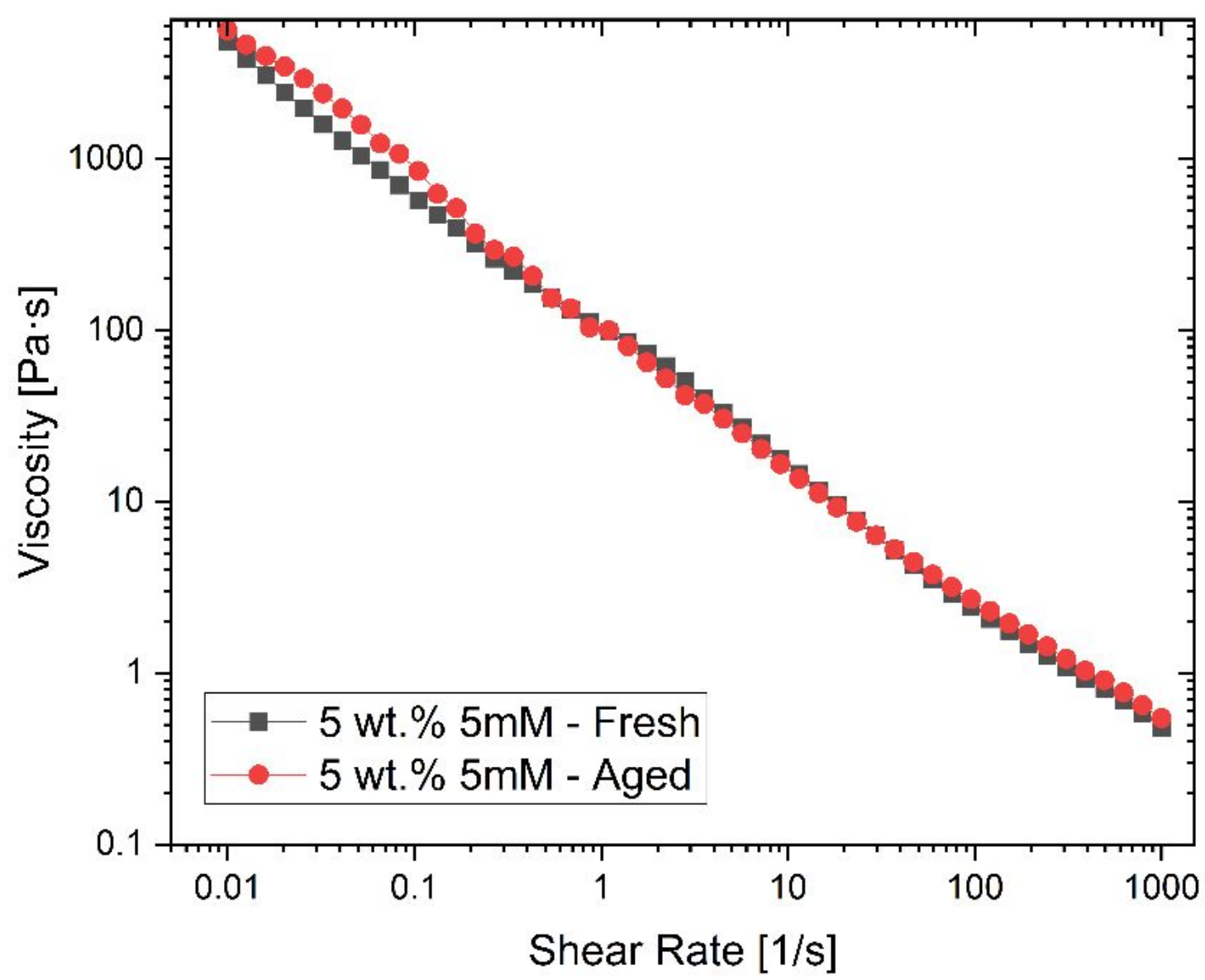

Figure S3: Steady shear viscosity as a function of shear rate for 5 wt.% particle-stabilized emulsions with 5 mM salt concentration, comparing fresh and aged samples.

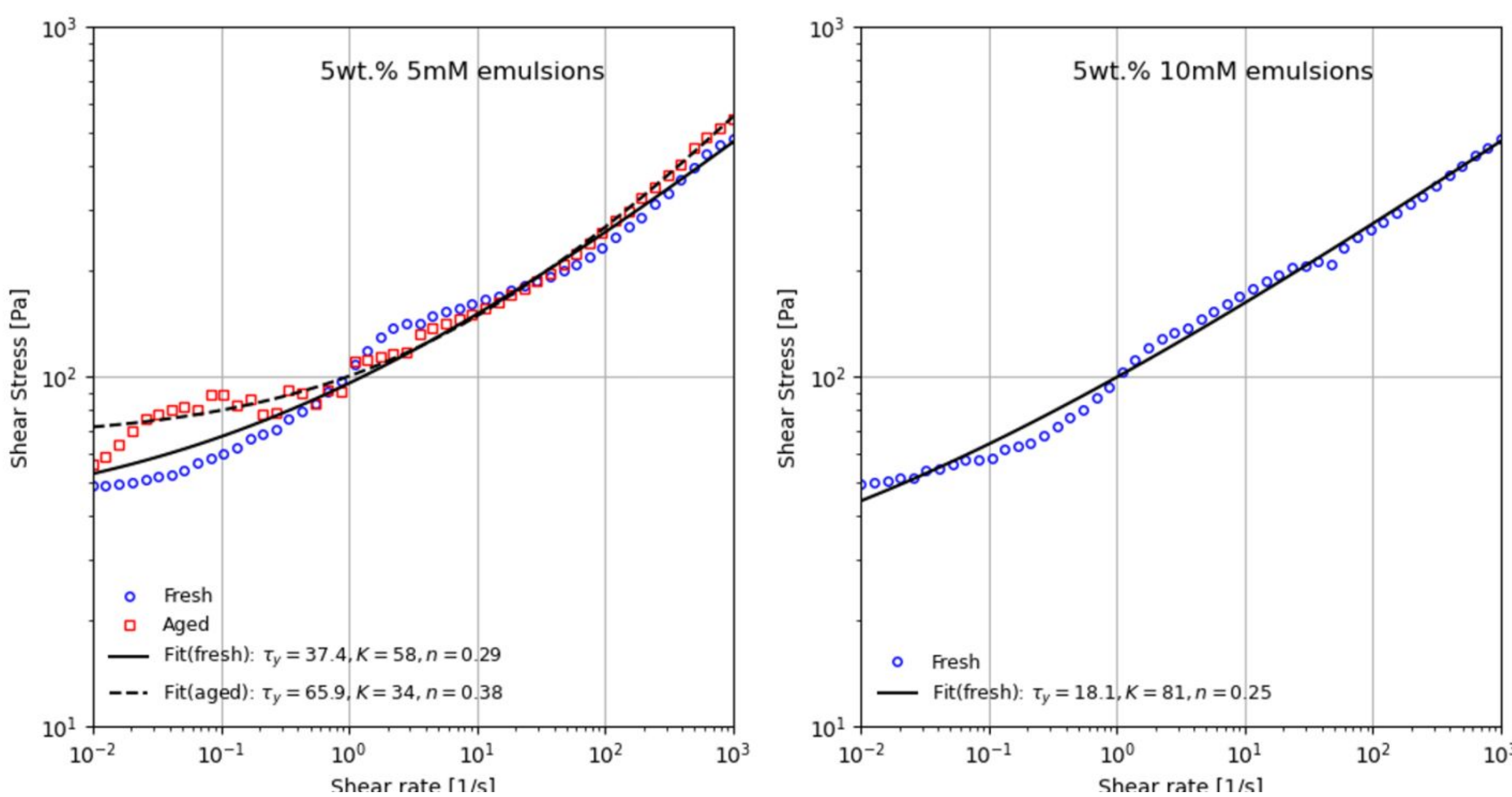

Figure S4: Shear stress as a function of shear rate for 5 wt.% particle-stabilized emulsions with 5 mM and 10 mM salt concentration. For the 10 mM system, only the fresh sample was measured

due to phase separation for the aged sample. The data has been plotted as log-log and was fitted to the Herschel-Bulkley model, giving the yield stress value (τ_y), the consistency index C, and the flow index n.

4. Small-angle X-ray scattering

   a. BLG particle dispersions

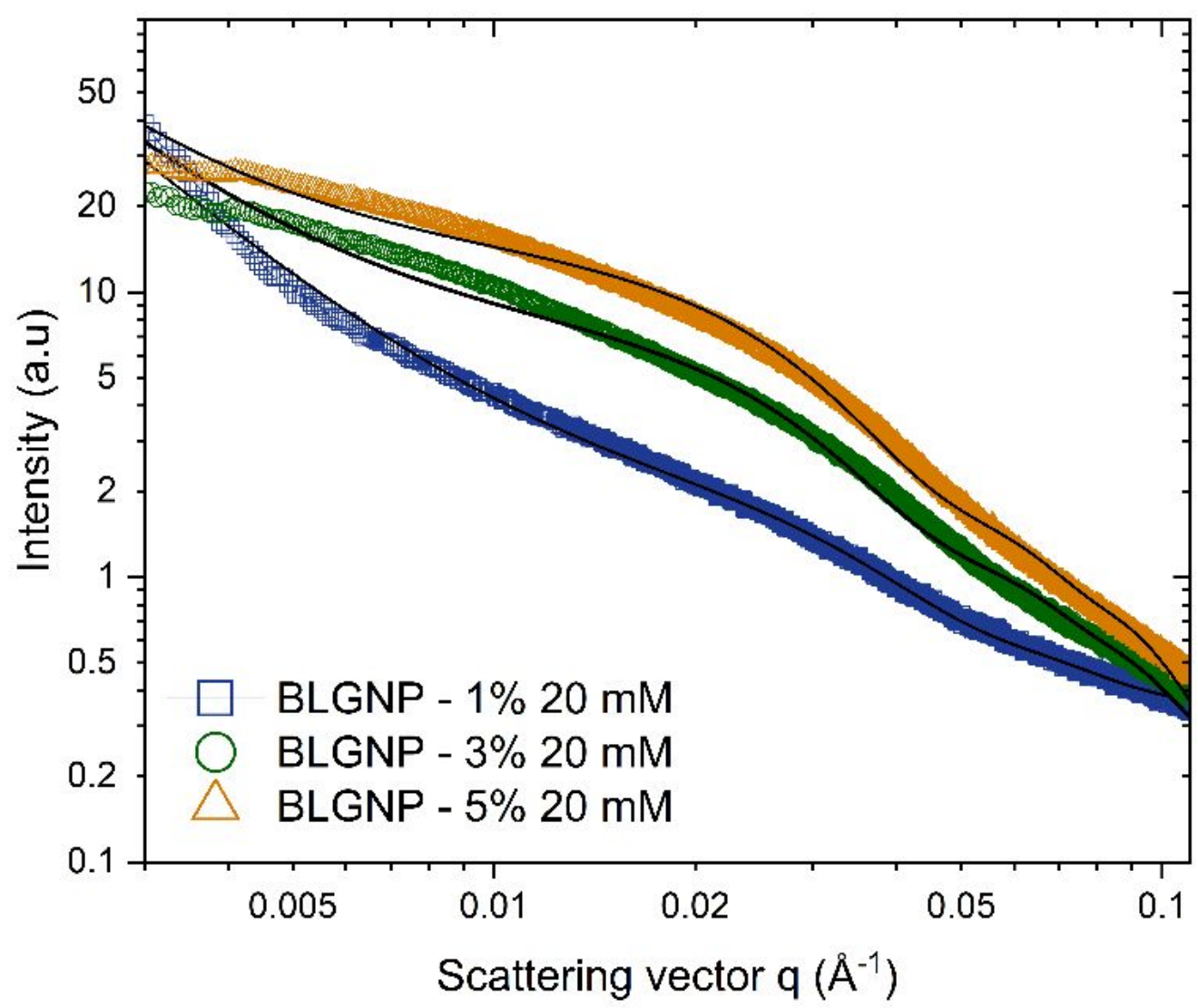

Figure S5: SAXS data for 1,3 and 5 wt.% nanoparticle dispersions with 20 mM salt concentration, showing Intensity vs scattering vector q measured at room temperature (25 ˚C). The data were fitted using a Guinier-Porod model combined with an ellipsoidal model with Hayter MSA as the structure factor. The coloured symbols represents the experimental data and the black line indicates the model fits

Table S2: Selected structural parameters deduced from the fits to the data for BLG particle dispersions with 1,3 and 5 wt.% particles and different salt concentrations (0, 5, 10 and 20 mM)

measured at room temperature (25 °C). Here Rp and Req are the average polar and equatorial radii of the BLGNP particles.

Sample name	Rp (Å)	Req (Å)
BLGNP/1/5	9.8± 1	122.1± 1.
BLGNP/3/5	10.4± 1	59.2 ± 1
BLGNP/5/5	10.1 ± 1	62.3 ± 1
BLGNP/1/10	13.2 ± 1	76.0 ± 1
BLGNP/3/10	13.6 ± 1	88.0 ± 1
BLGNP/5/10	12.1 ± 1	95.3± 1
BLGNP/1/20	20.5 ± 1	88.0 ± 1
BLGNP/3/20	17.9± 1	104.0 ± 1
BLGNP/5/20	17.8 ± 1	105.0 ± 1

b. High internal phase Pickering emulsions

Table S3: Model parameters for BLG particles in emulsions with 2-5 wt.% particles in 0-20 mM aqueous solution prepared with 75 wt.% dispersed phases. Here Rp and Req are the polar and equatorial radii of the Pickering particles, and the shell thickness defines the interface layer of oil

droplets. The model particle charge was 7.7 e- and dielectric constant for the dispersion medium was 71. The errors listed were calculated through a variation analysis using different starting values for the model parameters. Note that these errors are larger than the intrinsic standard deviations produced by SasView (< ± 0.5 Å).

**Sample name**	**Rp (Å)**	**Req (Å)**	**Shell thickness (Å)**
BLGNPem_2pt_0mM_75pt_Fresh	28.0 ± 1	264.0 ± 2	95.2 ± 1
BLGNPem_3pt_0mM_75pt_Fresh	18.3 ± 1	265.0 ± 2	92.9 ± 1
BLGNPem_4pt_0mM_75pt_Fresh	22.5 ± 1	119.1 ± 1	99.2 ± 1
BLGNPem_5pt_0mM_75pt_Fresh	19.8 ± 1	95.8 ± 1	101.8 ± 1
BLGNPem_2pt_5mM_75pt_Fresh	34.0 ± 1	129.2 ± 1	101.9 ± 1
BLGNPem_3pt_5mM_75pt_Fresh	35.1 ± 1	73.2 ± 1	80.1 ± 1
BLGNPem_4pt_5mM_75pt_Fresh	21.6 ± 1	87.4 ± 1	97.0 ± 1
BLGNPem_5pt_5mM_75pt_Fresh	19.8 ± 1	79.0 ± 1	92.1 ± 1
BLGNPem_2pt_10mM_75pt_Fresh	29.9 ± 1	100.9 ± 1	114.1 ± 1
BLGNPem_3pt_10mM_75pt_Fresh	30.5 ± 1	90.4 ± 1	124.3 ± 1
BLGNPem_4pt_10mM_75pt_Fresh	33.8 ± 1	79.6 ± 1	115.6 ± 1
BLGNPem_5pt_10mM_75pt_Fresh	33.4 ± 1	85.3 ± 1	107.5 ± 1
BLGNPem_3pt_20mM_75pt_Fresh	28.7 ± 1	96.7 ± 1	135.1 ± 1
BLGNPem_4pt_20mM_75pt_Fresh	31.1 ± 1	86.1 ± 1	140.7 ± 1

BLGNPem_5pt_20mM_75pt_Fresh	30.8 ± 1	89.0 ± 1	149.7 ± 1
